# Traumatic Intracranial Vascular Lesions in Adults Undergoing Vascular Imaging After Traumatic Brain Injury: A Systematic Review and Meta-Analysis

**DOI:** 10.3390/medicina62101830

**Published:** 2026-09-23

**Authors:** Iulia T. Lupascu, Costin A. Minoiu, Bogdan V. Popa, Corina Homentcovschi, Lucian M. Florescu, Sorin Hostiuc

**Affiliations:** 1 Department of Legal Medicine and Bioethics, “Carol Davila University of Medicine and Pharmacy” Bucharest, 050474 Bucharest, Romania; iulia-tatiana.lupascu@drd.umfcd.ro; 2 Clinical Emergency Hospital Bucharest, 014461 Bucharest, Romania; 3 Department of Radiology, “Carol Davila University of Medicine and Pharmacy” Bucharest, 050474 Bucharest, Romania; 4 Department of Medical Semiology “Carol Davila University of Medicine and Pharmacy” Bucharest, 050474 Bucharest, Romania; corina.homentcovschi@umfcd.ro; 5 Clinical Hospital Coltea, 030167 Bucharest, Romania; 6 Department of Radiology, “University of Medicine and Pharmacy of Craiova”, 200349 Craiova, Romania; lucian.florescu@umfcv.ro; 7 Clinical Emergency Hospital Craiova, 200642 Craiova, Romania; 8 The National Institute of Forensic Medicine “Mina Minovici”, 077160 Bucharest, Romania; sorin.hostiuc@umfcd.ro; 9 Department of Legal Medicine and Bioethics, Faculty of Dental Medicine, “Carol Davila University of Medicine and Pharmacy”, 020021 Bucharest, Romania

**Keywords:** traumatic brain injury, traumatic intracranial vascular lesions, intracranial vascular lesions, pseudoaneurysm, venous sinus thrombosis, arterial dissection, carotid–cavernous fistula, arteriovenous fistula

## Abstract

*Background and Objectives:* Traumatic intracranial vascular lesions are rare but severe complications of traumatic brain injury (TBI) and include arterial and venous injuries such as arterial dissections, pseudoaneurysms, arterial occlusions, venous sinus thrombosis, arteriovenous fistulas, and carotid–cavernous fistulas. Despite their clinical significance, the available literature remains limited and heterogeneous. This systematic review and meta-analysis aimed to evaluate the diagnostic yield of vascular imaging, spectrum, and imaging modalities of traumatic intracranial vascular lesions in adults with TBI. *Materials and Methods:* A systematic literature search was conducted from database inception to 31 August 2026 in PubMed, Web of Science Core Collection, Scopus, and the Cochrane Library (CENTRAL) according to the PRISMA 2020 guidelines. Cohorts of patients who underwent vascular imaging after TBI were eligible. When the full text was available, the denominator was the number of patients imaged with a modality able to detect the lesion in question and the numerator was reported at patient level for intracranial lesions. The primary analyses were restricted to cohorts with an explicit adult age criterion or a separately reported adult subgroup; cohorts with a pediatric fraction that could not be separated entered a sensitivity analysis only. Data extraction and quality assessment using the Newcastle–Ottawa Scale and the Joanna Briggs Institute checklist for prevalence studies were performed independently by two reviewers. Proportions were pooled with a logit-transformed random-effects model with restricted maximum-likelihood estimation and prediction intervals, with Freeman–Tukey and generalized linear mixed models as sensitivity analyses. *Results:* Nineteen studies with 3542 imaged patients were included, eight of them adult cohorts with 2021 patients. In adult cohorts, the pooled diagnostic yield was 11.0% (95% CI, 2.3–39.8%; four cohorts) for dural venous sinus thrombosis, 4.8% (95% CI, 1.1–18.4%; three cohorts) for pseudoaneurysm, 1.1% (95% CI, 0.0–20.8%; two cohorts) for arteriovenous fistula, and 0.7% (95% CI, 0.2–3.1%; three cohorts) for carotid–cavernous fistula, with I^2^ between 74.7% and 96.2% and prediction intervals spanning most of the possible range; arterial dissection (7.0%) and arterial occlusion (5.8%) were reported by a single adult cohort and were not pooled. The proportion of patients with any lesion was not pooled, because the seven cohorts that imaged every patient differed in whether and how the venous compartment was assessed; it ranged from 1.5% to 56.9% by cohort. Adding the eleven adult-dominant cohorts gave 17.5% for sinus thrombosis and 4.5% for pseudoaneurysm. In exploratory univariable meta-regressions for sinus thrombosis, trauma mechanism, imaging modality, and study design were each associated with part of the heterogeneity and were confounded with one another in these cohorts. *Conclusions:* Every estimate describes the yield of vascular imaging in adults selected for it, rests on two-to-four small cohorts, and carries very low certainty; the prediction intervals show that a new cohort may fall far from the pooled value, and none of the estimates can be read as the frequency of these lesions in TBI in general.

## 1. Introduction

Traumatic brain injury (TBI) represents a public health concern and a leading cause of death and disability across all ages. In low- and middle-income countries, the main etiology of TBI is represented by road traffic accidents, while in high-income countries, the number of TBIs among elderly people is increasing, mainly due to falls, underlining an epidemiological change [1].

Traumatic cerebrovascular injury (TCVI) is a consequence of both isolated neurotrauma and polytrauma and it is associated with high morbidity and mortality. TCVI is estimated to occur in 0.8 to 1.8% of TBI cases and can be subdivided into blunt or penetrating injury to intracranial or extracranial vascular structures [2].

Blunt trauma mechanisms include hyperextension with contralateral rotation of the head, direct vascular blow, and intraoral trauma, which can result in vessel dissection or direct laceration from bony fracture, while penetrating injuries imply a projectile or non-projectile object that pierces the body [3].

The main imaging techniques used in clinical practice for the diagnosis of TCVI include conventional angiography, computed tomographic angiography (CTA), and magnetic resonance angiography (MRA). For specific lesions such as post-traumatic venous sinus thrombosis, contrast-enhanced CTV or a magnetic resonance imaging (MRI)/magnetic resonance venography (MRV) can be used [4]. Even though conventional angiography represents the gold standard for the detection of vascular lesions, CTA is used as first-line imaging in clinical practice [5,6].

While TCVI consists of both extracranial and intracranial vascular lesions, literature data has focused mostly on extracranial injuries, particularly blunt carotid and vertebral artery injuries, with limited data on intracranial lesions.

Traumatic intracranial vascular lesions include arterial dissections, traumatic aneurysms (pseudoaneurysms), arterial occlusions, venous sinus thrombosis, and arteriovenous fistulas, including carotid–cavernous fistula [7]. To our knowledge, data on this topic are scarce and heterogeneous, without dedicated screening recommendations being universally adopted.

The purpose of this meta-analysis was to systematically review the literature on traumatic intracranial vascular lesions, assessing their epidemiology, lesion spectrum, and imaging modalities in order to address the heterogeneity and limited availability of data in this field.

## 2. Materials and Methods

### 2.1. Study Design

This study was conducted as a systematic review and meta-analysis of published studies evaluating traumatic intracranial vascular injuries, in accordance with the PRISMA 2020 guidelines. The completed PRISMA 2020 checklist is provided as Supplementary Material (Table S8). The review protocol was registered on the Open Science Framework (OSF Registration: osf.io/thjxp).

### 2.2. Search Strategy

A systematic literature search was conducted in PubMed, Web of Science Core Collection, Scopus, and the Cochrane Library (CENTRAL) from database inception to 31 August 2026, with no date, language, or age restriction. PubMed was searched with six documented queries, each combining a trauma block with one lesion block, excluding only the editorial, letter, comment, and news publication types. The equivalent native syntax was used for the other three databases on the same date. The complete queries, with the number of records returned by each, are given in Supplementary Materials. In addition, the reference lists of relevant articles and published reviews were manually screened to identify eligible studies. This study’s selection process is shown in the PRISMA flow diagram (Figure 1).

### 2.3. Eligibility Criteria

Studies were included if they met the following features: study population consisting of either men, women, or both; an adult population, defined for the primary analyses as an explicit inclusion criterion of 18 years or over, an age range starting at 18 years, or a separately reported adult subgroup; cohorts with a mean or median age above 18 years but with a pediatric or adolescent fraction that cannot be separated, or with no stated age criterion, were classed as adult-dominant and entered the sensitivity analyses only; written in the English language; full text available, so that reports whose counts could be read only from the abstract were not eligible for any quantitative analysis; a denominator defined by the number of patients imaged with a modality able to detect the lesion in question; and a numerator reported at patient level for intracranial lesions. Studies were excluded if they were case reports, reviews, or commentaries without original data; pediatric cohorts, or cohorts in which adults were not the majority; if the cohort was assembled on the presence of the lesion, with a denominator not defined by imaging; if only cervical or extracranial vessels were involved; or if counts were available only at the lesion level.

### 2.4. Data Extraction

Data extraction was performed independently by two of the authors using a standardized form in Microsoft Excel. Extracted variables included:

Study characteristics: author, year, country, study design, and number of patients;

Population characteristics: trauma type (blunt or penetrating) and patient demographics;

Diagnostic methods: imaging modality used to detect vascular injuries (CTA/DSA/CTV/MRA);

Outcomes: prevalence of traumatic intracranial vascular lesions and specific subtypes, such as arterial dissection, pseudoaneurysm, arterial occlusion, venous sinus thrombosis, arteriovenous fistula, and carotid–cavernous fistula;

Data for meta-analysis: number of patients presented with each lesion and total sample size.

All counts used in the meta-analysis were verified at the patient level against the source publications. Counts available only at the lesion level were not entered; for cohorts that reported their subtype counts as lesions, only the counts of patients with any lesion were used, as shown in the cohort-by-cohort description of Section 3.3.1. A zero was entered when the source listed the lesion among the categories it assessed and reported none; a lesion type absent from a source’s classification was recorded as not reported, and that study did not enter the analysis of that outcome. Only lesions that the source located intracranially were counted. A cohort assembled on the presence of the lesion, or whose denominator was not the number of patients imaged, was excluded; a cohort in which the modality able to detect a given lesion was applied to a subset selected on clinical suspicion was excluded from the analysis of that lesion. The application of these rules to each included and excluded study is reported in Section 3.2.

### 2.5. Statistical Analysis

The outcome of every analysis is the diagnostic yield of vascular imaging, defined as the number of patients with an imaging-confirmed traumatic intracranial vascular lesion divided by the number of patients who underwent the relevant vascular imaging study in each cohort. Six lesion-specific outcomes were analyzed: arterial dissection, pseudoaneurysm, arterial occlusion, dural venous sinus thrombosis, arteriovenous fistula, and carotid–cavernous fistula. The primary analysis of each outcome used the adult cohorts; a prespecified sensitivity analysis added the adult-dominant cohorts. The proportion of patients with any lesion was not pooled: the seven cohorts in which every patient underwent angiographic imaging differed in whether the venous compartment was assessed at all, on the venous phase of an arterial study or by dedicated venography, and in which lesion categories the source counted, so their yields were reported cohort by cohort (Table S5). Pooled estimates were obtained with a random-effects model applied to logit-transformed proportions, with between-study variance estimated by restricted maximum likelihood. The logit scale was adopted because the Freeman–Tukey double arcsine transformation returns a pooled estimate on a transformed scale whose back transformation depends on an assumed sample size and becomes unstable when the observed proportions approach zero. Two sensitivity analyses were run for every pooled outcome: a Freeman–Tukey double arcsine model with harmonic mean back transformation, and a binomial normal generalized linear mixed model with a random study intercept, retaining zero-event studies without continuity correction. An outcome reported by a single adult cohort was given as that cohort’s proportion with its Wilson interval and was not pooled. Heterogeneity was reported from the same logit model that produced the estimates, as I^2^, tau squared, and Cochran Q, and a prediction interval accompanied every pooled estimate, since with substantial between-study variance, the confidence interval around the mean says little about the value expected in a new cohort; with two-to-four cohorts, the absence of detectable heterogeneity was reported as such and was not read as evidence that the cohorts were comparable. Moderators were examined in the sensitivity sets only, one at a time, by univariable meta-regression with a single residual between-study variance; the proportion of heterogeneity associated with each moderator in its own model was reported as exploratory, and the moderators were not ranked against one another, because they were confounded in the available cohorts. Leave-one-out analysis was run for every pooled outcome with at least three cohorts. Publication bias was not tested formally, since no primary analysis reached the ten studies conventionally required. All analyses were run in R 4.3.3 with metafor 4.4.0. 

### 2.6. Risk of Bias/Quality Assessment

The methodological quality of the included studies was assessed by two reviewers using the Newcastle–Ottawa Scale (NOS) for observational studies. The scale evaluated selection, comparability, and outcome assessment. Disagreements were resolved by consensus. Each study was additionally appraised with the Joanna Briggs Institute checklist for prevalence data, which is the instrument designed for prevalence questions, and the Joanna Briggs judgement was used as the risk-of-bias input to the GRADE assessment. The results are presented in Tables S1 and S2.

The certainty of the evidence for each pooled estimate was rated with the GRADE approach adapted for questions of prevalence. Bodies of evidence composed exclusively of observational prevalence studies started at moderate certainty and were downgraded for risk of bias, inconsistency, indirectness, and imprecision. GRADE was applied to the six lesion-specific outcomes of the primary analysis, listed in Table S3 Certainty was very low for all six, because every cohort was assembled on an imaging indication, because most cohorts were retrospective, because heterogeneity exceeded 75% in three of the four pooled outcomes, because no outcome rested on more than four cohorts, and because two outcomes rested on a single cohort. The domain-by-domain ratings are presented in Table S3.

## 3. Results

### 3.1. Selection Process (PRISMA Guidelines)

This study’s search and selection process were conducted according to the Preferred Reporting Items for Systematic Reviews and Meta-Analyses (PRISMA) guidelines (PRISMA flowchart available in Figure 1).

A total of 11,296 records were identified through database searching: 3887 from six PubMed queries, 2335 from Web of Science, 5034 from Scopus, and 40 from Cochrane CENTRAL. Removing 4206 duplicates left 7090 records for title and abstract screening, of which 6732 were excluded. Full text was sought for 358 reports and obtained for 268. Of these, 249 were excluded, most often because the cohort was defined by the lesion (96 reports) or because the lesions were cervical or extracranial (71 reports). Nineteen studies entered the analyses (Table 1): eight adult cohorts with 2021 patients, which carry the primary analyses, and eleven adult-dominant cohorts with 1521 patients, which enter the sensitivity analyses. Ten further reports identified by the updated search could not be retrieved in full text although their abstract state counts; they are described in Table S7 and are not pooled. Exclusion at the eligibility stage also applied to the series of Kobata et al. [8], whose denominator comprises the 33 patients with confirmed traumatic cerebrovascular injury among 1966 admissions, and which also includes pediatric patients.

### 3.2. Study Characteristics

A total of 3542 imaged patients were included across nineteen studies. Eleven cohorts were penetrating trauma, seven blunt and one mixed, and fourteen were retrospective. Imaging was digital subtraction angiography alone in six cohorts, computed tomographic angiography alone in two, computed tomographic angiography with catheter angiography in four, computed tomographic angiography with venography in two, and dedicated venography in five. Sex was reported for 1436 patients, of whom 1104 (76.9%) were male and 332 (23.1%) were female. Mean age ranged from 26 to 47 years.

Four cohorts report their subtype counts as lesions, Bell et al. [12] (64 injuries in 47 patients), Meyer et al. [25] (47 lesions in 33 patients, 14 of whom had more than one), Bodanapally et al. [16] (28 lesions in 21 patients), and Mansour et al. [23] (37 arterial lesions in 26 patients), and contribute only their count of patients with any lesion. The 24 intracranial aneurysms of Bell et al. [12] were entered and the 17 extracalvarial ones were not; the fistulas of Ares et al. [19] were entered while their external and common carotid lesions were not. Vascular injuries were found in 7 of 26 patients in the series of Karanja et al. [17], three with a pseudoaneurysm and four with a vessel cut-off, a count stated at patient level and entered as such; patient-level figures are likewise used for Dawoud et al. [21], 28 of 63, and for Sirko et al. [27], 20 of 1310. Two studies did not meet the denominator rule and were excluded: the cohort of Achram et al. [28], whose denominator of 219 count admissions, since the angiograms reviewed were those of patients already known to carry an aneurysm, and that of Huang et al. [29], whose 112 patients were assembled on confirmed sinus thrombosis. Venography was performed in 45 of the 72 patients of Mansour et al. [23] on clinical suspicion, so that study is excluded from the venous sinus thrombosis analysis.

### 3.3. Meta-Analysis

#### 3.3.1. Overall Intracranial Traumatic Vascular Lesions

Seven cohorts applied angiographic imaging to every patient and reported the number of patients with at least one intracranial lesion, three adult cohorts and four adult-dominant ones (Table S5, Figure 2). Their protocols differ in the venous compartment: Lamanna et al. [24] performed CT venography in every patient; Meyer et al. [25] and Dawoud et al. [21] read the dural sinuses on the venous phase of an arterial study; Mansour et al. [23] performed venography in 45 of 72 patients on clinical suspicion; and Bodanapally et al. [16], Sirko et al. [27], and Karanja et al. [17] did not assess it. They also differ in what the source counted: Dawoud et al. [21] include nine patients with vasospasm and three with arterial transection, and Lamanna et al. [24] include seven with sinus impingement and six with transection, categories outside the six lesions of this review.

For these reasons, no pooled estimate of any lesion was computed. The yield by cohort was 50.6% (95% CI, 43.2–58.0%) for Lamanna et al. [24], 38.2% (95% CI, 26.5–51.4%) for Bodanapally et al. [16], and 1.5% (95% CI, 1.0–2.3%) for Sirko et al. [27] among the adult cohorts, and 56.9%, 45.2%, 44.4%, and 26.9% for Mansour et al. [23], Meyer et al. [25], Dawoud et al. [21], and Karanja et al. [17] The spread from 1.5% to 56.9% follows the extent of assessment and the indication for imaging in each series: Sirko et al. [27] angiographed consecutive head and neck wounds irrespective of an indication specific to brain injury, whereas the other cohorts imaged patients selected on a clinical or radiological indication.

#### 3.3.2. Subgroup Analysis: Traumatic Intracranial Arterial Dissection

One adult cohort reported intracranial arterial dissection at the patient level, Lamanna et al. [24], with 12 of 172 patients presenting 7.0% (95% CI, 4.0–11.8%) (Figure 3); no pooled estimate is computed for the primary analysis. In the sensitivity analysis adding Dawoud et al. [21] and Ahmed et al. [11], three cohorts with 259 patients and 21 events gave 8.3% (95% CI, 5.4–12.4%) with no detectable heterogeneity (I^2^ = 0%; Q = 1.06, df = 2; *p* = 0.59), a result that states the absence of detectable statistical heterogeneity across three small cohorts and nothing more. Three further cohorts (Bell et al. [12], Bodanapally et al. [16], and Mansour et al. [23]) report dissection only as a lesion count and are not entered.

**Figure 3:**
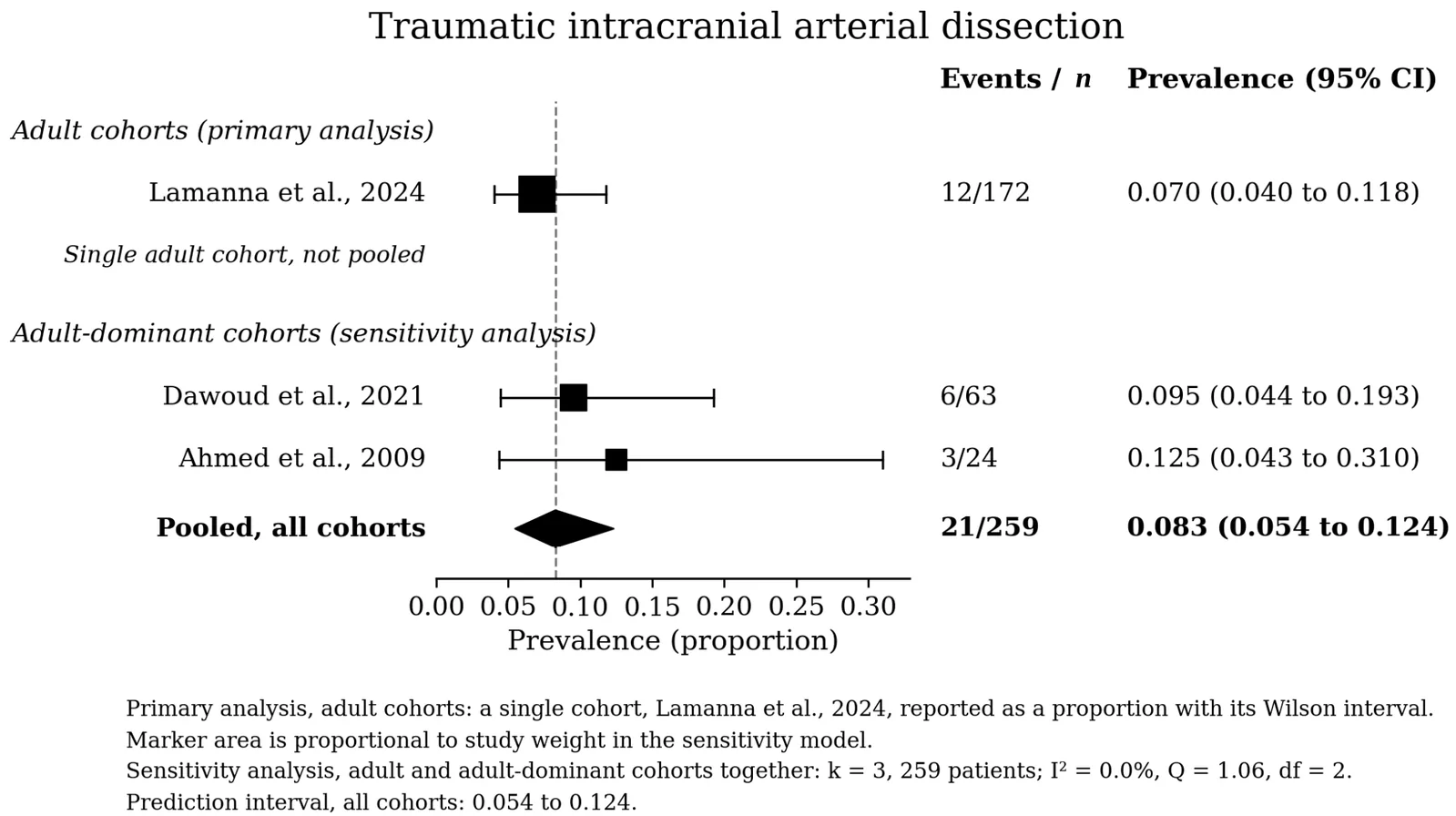
Forest plot of traumatic intracranial arterial dissection. Upper block, adult cohorts (primary analysis): a single cohort, Lamanna et al. [24]: 12 of 172, not pooled. Lower block, adult-dominant cohorts, Dawoud et al. [21] and Ahmed et al. [11]; the diamond is the sensitivity estimate on all three cohorts, 0.083 (95% CI, 0.054 to 0.124); I^2^ = 0%. Squares are study estimates with areas proportional to weight and horizontal lines are 95% Wilson intervals.

#### 3.3.3. Subgroup Analysis: Traumatic Intracranial Pseudoaneurysm

Three adult cohorts with 1669 patients reported 51 patients with a traumatic intracranial pseudoaneurysm (Figure 4), giving a pooled diagnostic yield of 4.8% (95% CI, 1.1–18.4%) with high heterogeneity (I^2^ = 96.2%; Q = 57.32, df = 2; *p* < 0.001) and a prediction interval ranging from 0.3 to 49.0%. The three estimates were 1.1% for Sirko et al. [27] 7.0% for Lamanna et al. [24], and 12.8% for Bell et al. [12]; without the results of Sirko et al. [27], the estimate is 9.8% (95% CI, 5.3–17.3%). Sensitivity analyses gave 5.8% with the Freeman–Tukey model and 4.7% with the generalized linear mixed model. Adding the six adult-dominant cohorts, nine cohorts with 2538 patients and 73 events gave 4.5% (95% CI, 2.2–9.2%; I^2^ = 88.7%; prediction interval: 0.5–30.7%). Formal testing for publication bias was not performed, since both sets fall below the conventional threshold of ten studies.

**Figure 4:**
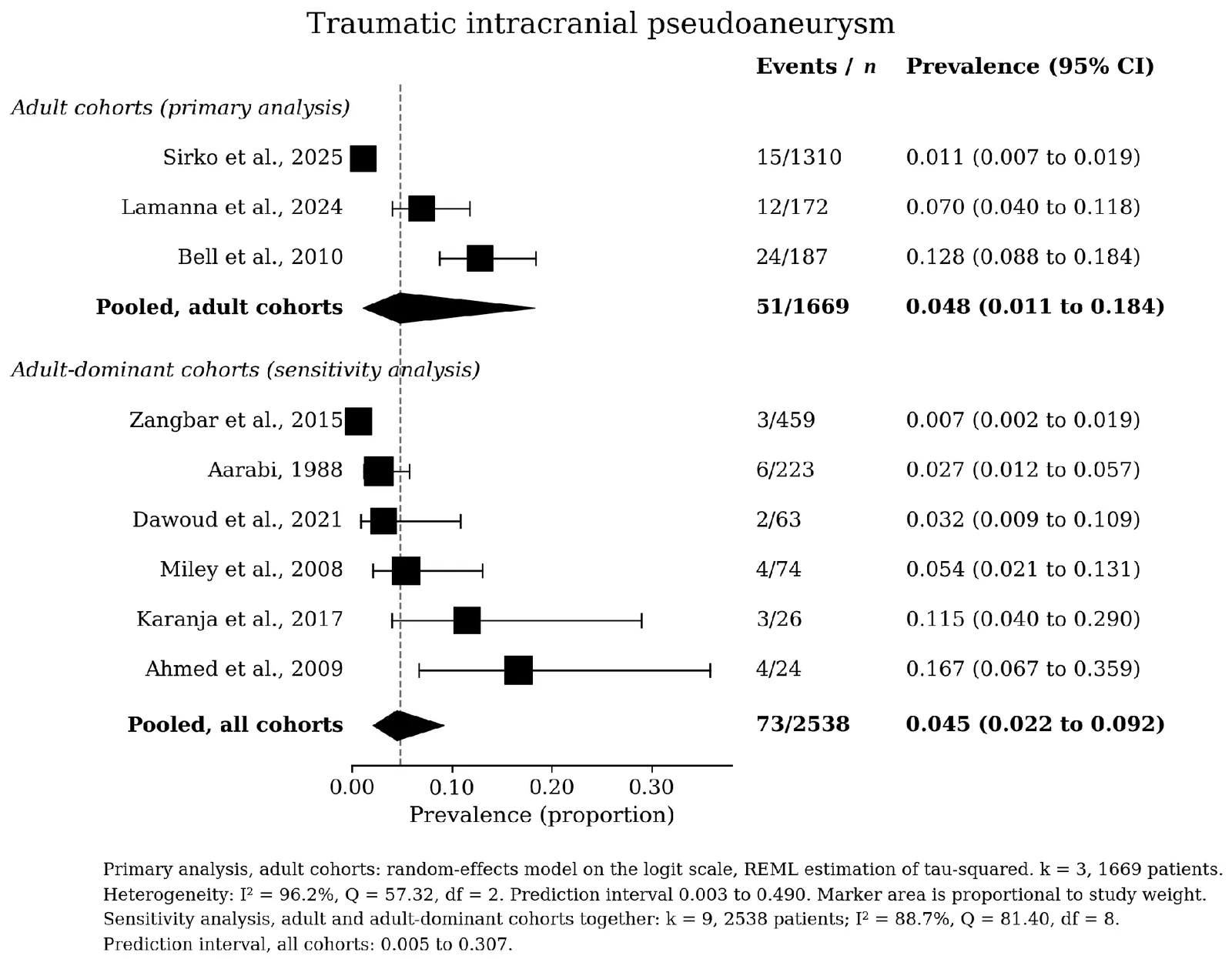
Forest plot of traumatic intracranial pseudoaneurysm. Upper block, adult cohorts (primary analysis), three studies and 1669 patients (Sirko et al. [27], Lamanna et al. [24], Bell et al. [12]); the upper diamond is the pooled estimate, 0.048 (95% CI, 0.011 to 0.184); I^2^ = 96.2%. Lower block, adult-dominant cohorts, Zangbar et al. [15], Aarabi et al. [9], Dawoud et al. [21], Miley et al. [10], Karanja et al. [17], and Ahmed et al. [11]; the lower diamond is the sensitivity estimate on all nine cohorts, 0.045 (95% CI, 0.022 to 0.092); I^2^ = 88.7%. Six cohorts were imaged with digital subtraction angiography and three with CT angiography.

#### 3.3.4. Subgroup Analysis: Traumatic Intracranial Arterial Occlusion

One adult cohort reported intracranial arterial occlusion at the patient level, Lamanna et al. [24], with 10 of 172 patients presenting 5.8% (95% CI, 3.2–10.4%) (Figure 5); no pooled estimate is computed for the primary analysis. In the sensitivity analysis adding Karanja et al. [17] and Dawoud et al. [21], three cohorts with 261 patients and 28 events gave 12.7% (95% CI, 5.3–27.5%) with substantial heterogeneity (I^2^ = 80.3%; Q = 12.05, df = 2; *p* = 0.002) and a prediction interval ranging from 2.5 to 45.7%. Occlusion is reported only as a lesion count by three further cohorts (Meyer et al. [25], Bodanapally et al. [16], and Mansour et al. [23]), and it is absent from the lesion categories assessed by Bell et al. [12] and Sirko et al. [27].

**Figure 5:**
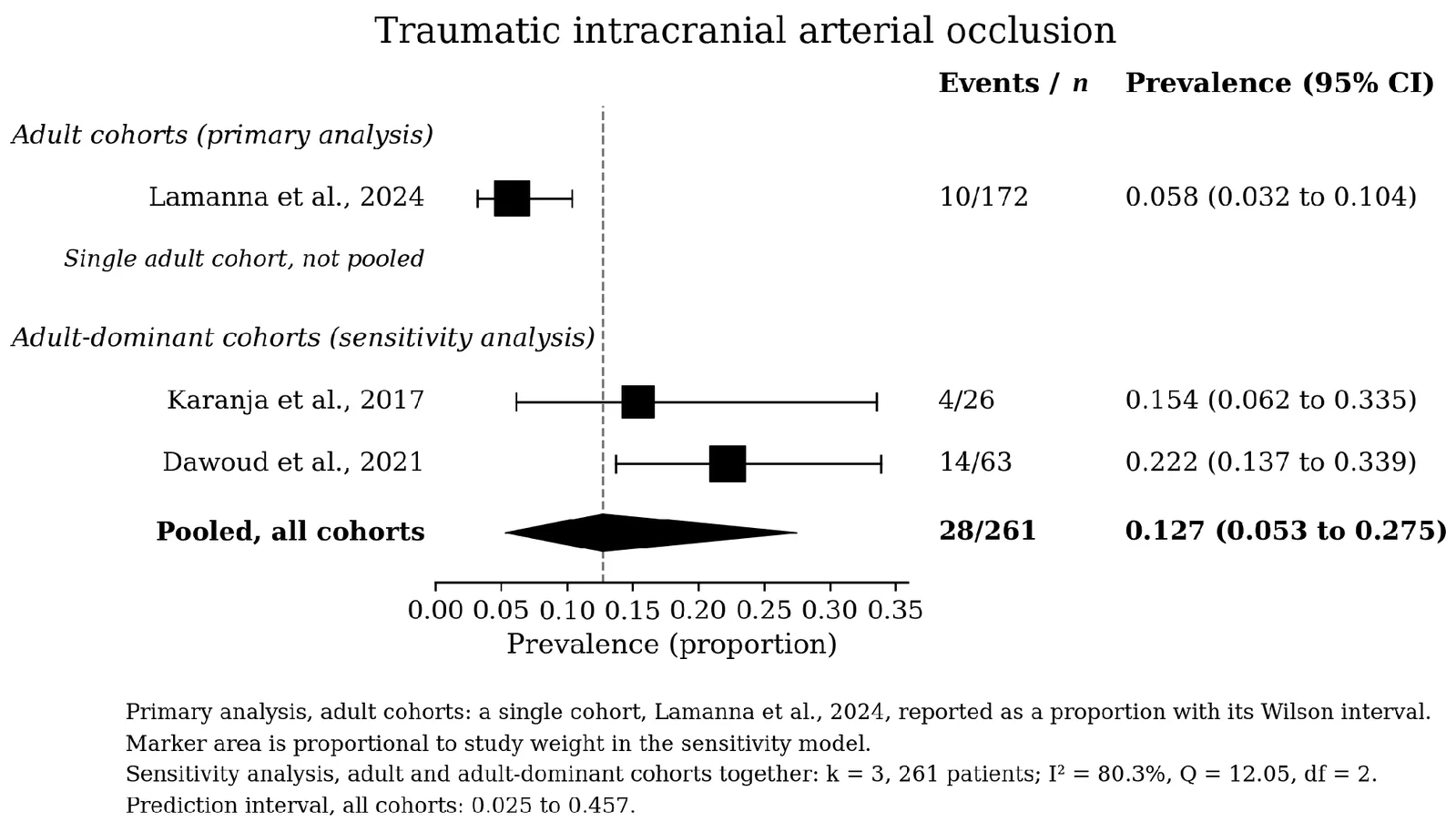
Forest plot of traumatic intracranial arterial occlusion. Upper block, adult cohorts (primary analysis): a single cohort, Lamanna et al. [24].: 10 of 172, not pooled. Lower block, adult-dominant cohorts, including the four vessel cut-offs reported by Karanja et al. [17] and Dawoud et al. [21]; the diamond is the sensitivity estimate on all three cohorts, 0.127 (95% CI, 0.053 to 0.275); I^2^ = 80.3%.

#### 3.3.5. Subgroup Analysis: Traumatic Intracranial Arteriovenous Fistula

Two adult cohorts with 1366 patients reported 6 patients with an intracranial arteriovenous fistula (Figure 6), 3 of 56 for Ares et al. [19] and 3 of 1310 for Sirko et al. [27], giving a pooled diagnostic yield of 1.1% (95% CI, 0.0–20.8%) with I^2^ = 93.3% (Q = 14.97, df = 1; *p* < 0.001) and a prediction interval ranging from 0.0 to 69.9%, an estimate that carries almost no information beyond the two cohort values. Sensitivity analyses gave 1.5% and 0.9%. Adding Dawoud et al. [21], an explicit zero among 63, and Ahmed et al. [11], 1 of 24, four cohorts gave 1.4% (95% CI, 0.3–7.2%; I^2^ = 77.1%). The eight dural arteriovenous fistulas in Meyer et al.’s [25] results are a lesion count and are not entered.

**Figure 6:**
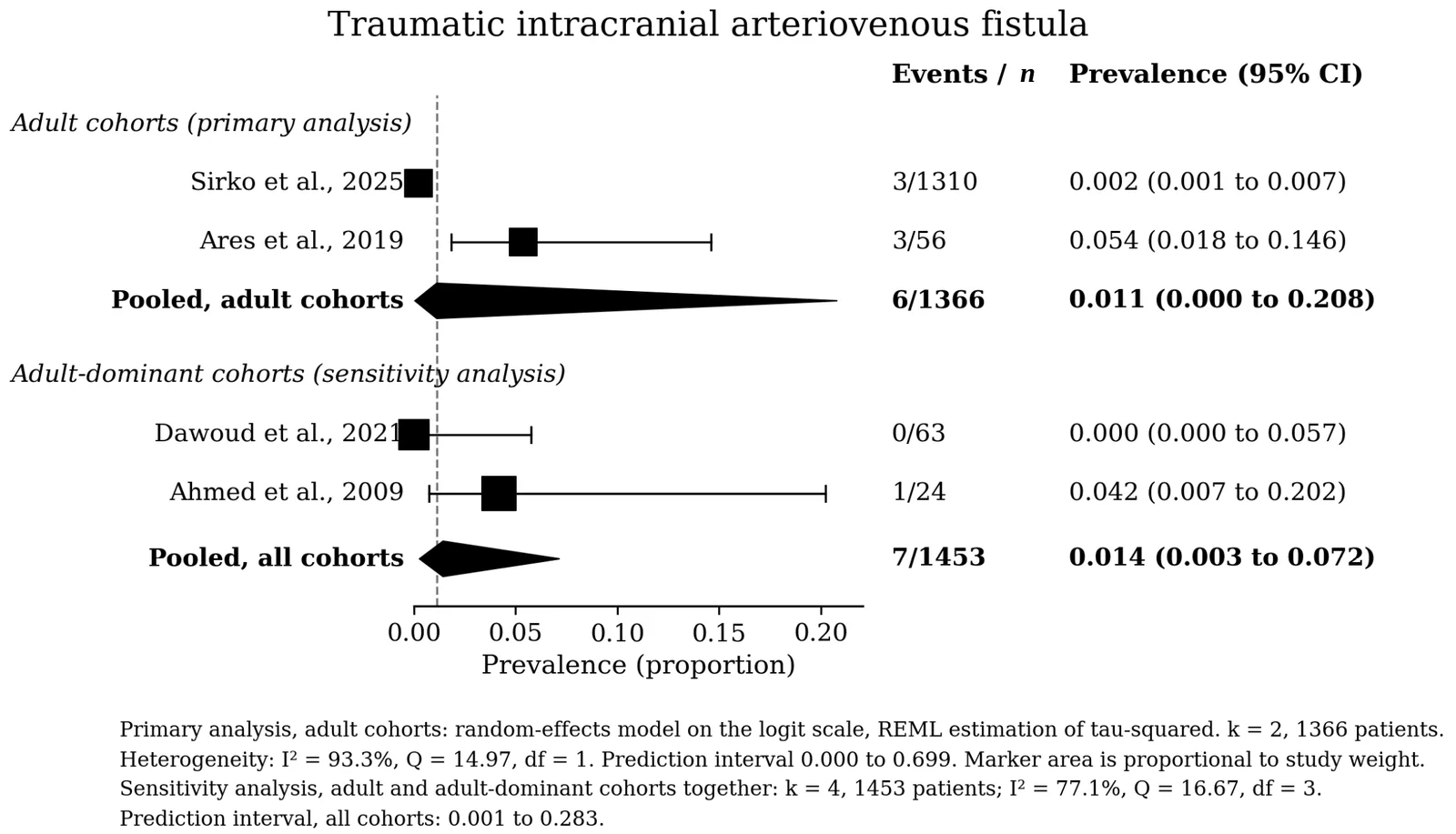
Forest plot of traumatic intracranial arteriovenous fistula. Upper block, adult cohorts (primary analysis), two studies and 1366 patients (Sirko et al. [27] and Ares et al. [19]); the upper diamond is the pooled estimate, 0.011 (95% CI, 0.000 to 0.208); I^2^ = 93.3%. Lower block, adult-dominant cohorts, one of which reported no events (Dawoud et al. [21] and Ahmed et al. [11]); the lower diamond is the sensitivity estimate on all four cohorts, 0.014 (95% CI, 0.003 to 0.072); I^2^ = 77.1%.

#### 3.3.6. Subgroup Analysis: Traumatic Intracranial Venous Thrombosis

Four adult cohorts with 413 patients reported 51 patients with dural venous sinus thrombosis or occlusion (Figure 7), giving a pooled diagnostic yield of 11.0% (95% CI, 2.3–39.8%) with high heterogeneity (I^2^ = 95.1%; Q = 24.00, df = 3; *p* < 0.001) and a prediction interval ranging from 0.3 to 81.7%. The four estimates were 0.6% for Harrington et al. [22], 13.9% for McGuckin et al. [26], 15.7% for Lamanna et al. [24], and 36.7% for Rivkin et al. [14]; without Harrington et al.’s [22] results, the one adult cohort in which the sinuses were read on an arterial study alone, the estimate is 21.0% (95% CI, 10.7–37.3%). Sensitivity analyses gave 13.5% and 10.0%. Adding the six adult-dominant cohorts, ten cohorts with 1080 patients and 213 events gave 17.5% (95% CI, 10.8–27.2%; I^2^ = 90.4%; prediction interval 3.8–53.4%). The cohort of Mansour et al. [23] remains excluded, because venography was performed in 45 of its 72 patients on clinical suspicion. The exploratory univariable meta-regressions in the ten-cohort set are reported in Section 3.4.

**Figure 7:**
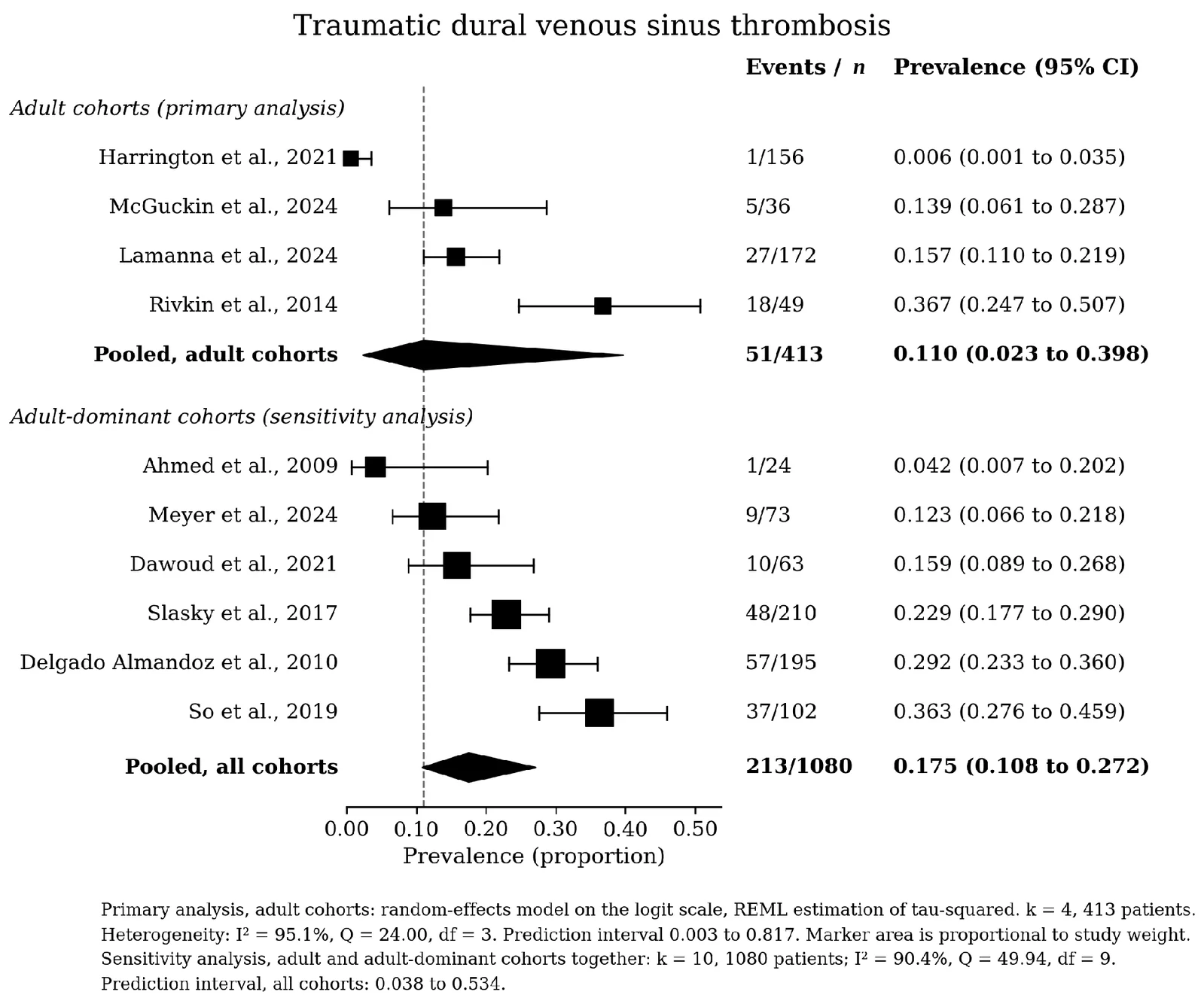
Forest plot of traumatic dural venous sinus thrombosis. Upper block, adult cohorts (primary analysis), four studies and 413 patients (Harrington et al. [22], McGuckin et al. [26], Lamanna et al. [24], and Rivkin et al. [14]); the upper diamond is the pooled estimate, 0.110 (95% CI, 0.023 to 0.398); I^2^ = 95.1%. Lower block, adult-dominant cohorts (Ahmed et al. [11], Meyer et al. [25], and Dawoud et al. [21]); the lower diamond is the sensitivity estimate on all ten cohorts, 0.175 (95% CI, 0.108 to 0.272); I^2^ = 90.4%. The four cohorts with the highest estimates, Rivkin et al. [14], So et al. [20], Delgado Almandoz et al. [13], and Slasky et al. [18], were imaged with dedicated CT venography after blunt trauma with a fracture adjacent to a dural sinus.

#### 3.3.7. Subgroup Analysis: Traumatic Intracranial Carotid–Cavernous Fistula

Three adult cohorts with 1638 patients reported 7 patients with a carotid–cavernous fistula (Figure 8), 2 of 156 for Harrington et al. [22], 2 of 1310 for Sirko et al. [27], and 3 of 172 for Lamanna et al. [24], giving a pooled diagnostic yield of 0.7% (95% CI, 0.2–3.1%) with I^2^ = 74.7% (Q = 7.80, df = 2; *p* = 0.020) and a prediction interval ranging from 0.0 to 9.7%. Sensitivity analyses gave 0.7% and 0.6%. Adding Mansour et al.’ [23] results, 1 of 72, four cohorts gave 0.8% (95% CI, 0.3–2.6%; I^2^ = 62.4%). The six carotid–cavernous fistulas of Meyer et al.’s [25] study and the three for Bodanapally et al. [16] are lesion counts and are not entered.

**Figure 8:**
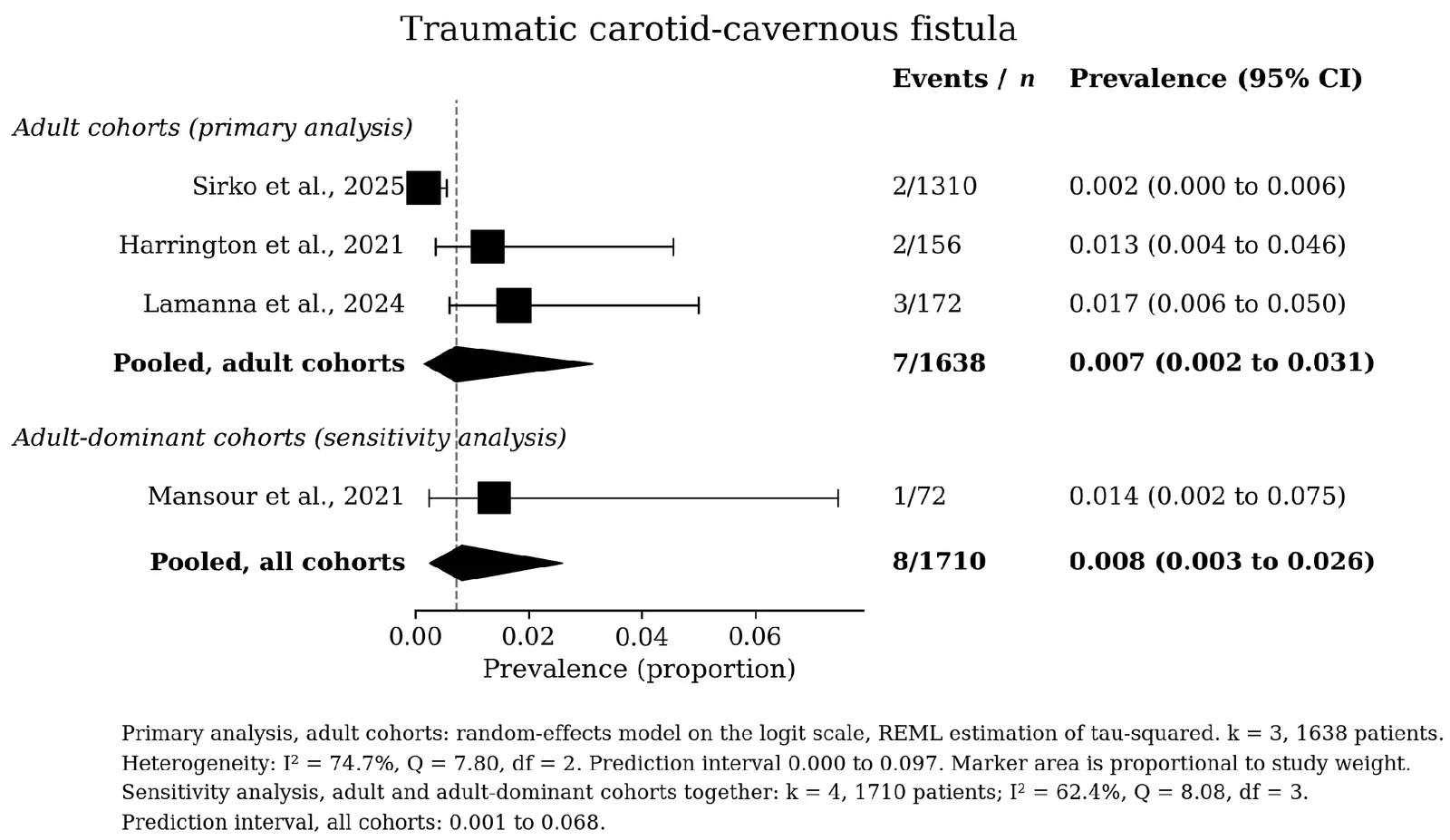
Forest plot of traumatic carotid–cavernous fistula, all penetrating trauma cohorts. Upper block, adult cohorts (primary analysis), three studies and 1638 patients (Sirko et al. [27], Harrington et al. [22], and Lamanna et al. [24]); the upper diamond is the pooled estimate, 0.007 (95% CI, 0.002 to 0.031); I^2^ = 74.7%. Lower block, the one adult-dominant cohort (Mansour et al. [23]); the lower diamond is the sensitivity estimate on all four cohorts, 0.008 (95% CI, 0.003 to 0.026); I^2^ = 62.4%.

Publication bias was not assessed formally for any outcome, since no primary analysis reached the ten studies conventionally required; funnel plots for the ten-cohort sinus thrombosis set and the nine-cohort pseudoaneurysm set are presented in the Supplementary Materials as Figures S1 and S2 and are descriptive only. Twenty-two of the reports that could not be retrieved are conference abstracts.

### 3.4. Subgroup Analyses

Moderators were examined in the sensitivity sets, which are the only ones with enough cohorts, one at a time, by univariable meta-regression with a single residual between-study variance; the results are exploratory and are given in full in Table S4. For dural venous sinus thrombosis (ten cohorts), blunt trauma was associated with a higher yield than penetrating trauma, 26.7% (95% CI, 19.9–35.0%; six cohorts) against 9.0% (95% CI, 2.9–24.8%; four cohorts), *p* = 0.008; dedicated venous imaging with a higher yield than an arterial study, 25.4% (95% CI, 18.6–33.7%; six cohorts) against 6.2% (95% CI, 1.6–21.1%; four cohorts), *p* = 0.006; and retrospective design with a higher yield than prospective design, 24.0% against 6.0%, *p* = 0.019. The proportion of heterogeneity associated with each moderator in its own univariable model was 72.7%, 53.7%, and 50.3%. These three figures come from separate models and cannot be added, compared, or read as independent contributions: five of the six blunt trauma cohorts used dedicated venous imaging and every penetrating-trauma cohort was assessed on an arterial study, so the moderators are confounded and the data cannot say which of them, if any, accounts for the difference. For pseudoaneurysm (nine cohorts), neither imaging modality (5.8% with digital subtraction angiography against 2.6% with CT angiography; *p* = 0.33) nor study design (*p* = 0.13), nor trauma mechanism (*p* = 0.11) was associated with the variation.

## 4. Discussion

The main focus of our study was a systematic review of the published literature to evaluate the proportion of traumatic intracranial vascular lesions.

In our analysis, a total of 3542 imaged patients were included. Sex was reported for 1436 patients, of whom 1104 were male and 332 were female, confirming a marked male predominance.

Sex distribution was reported primarily in civilian trauma cohorts, which included both male and female patients. Studies involving military trauma, which are likely to be predominantly male, did not report gender data and were excluded from this analysis. Therefore, the observed distribution is more representative of general civilian trauma populations. Our result is consistent with the literature data, which reports males being engaged more often in dangerous behavior and high-risk activities such as contact sports, alcohol consumption, substance abuse [30,31].

The mean age of the included populations ranged from approximately 26 to 47 years, with a weighted mean of 39.4 years across the twelve studies reporting age; two of these reported medians, so the weighted figure should be read as an approximation. This distribution is consistent with the predominance of high-energy and penetrating trauma in the included cohorts, which is more common in younger individuals [32].

The proportion of patients with any lesion could not be pooled. Among the seven cohorts that imaged every patient, the yield ranges from 1.5% in Sirko et al. [27] to 56.9% in Mansour et al. [23], and the range follows the extent of vascular assessment and the indication for imaging: the arterial-only series assembled on consecutive wounds sits at the bottom, and the series that assessed both compartments in patients selected on clinical or radiological grounds sit at the top. Three of these cohorts read the venous compartment in three different ways, by dedicated CT venography (Lamanna et al. [24]), by the venous phase of digital subtraction angiography (Meyer et al. [25]), and by the venous phase of CT angiography (Dawoud et al. [21]), and their numerators include lesion categories outside the six of this review, so their yields of 44% to 51% are three different measurements that happen to lie close together, and the proximity of the values is no evidence of a comparable protocol.

Population-based series report substantially lower incidences of traumatic cerebrovascular injury, intracranial and extracranial combined: around 0.8% in some studies [33], 1.6% in tertiary critical care, and 9.2% in patients with severe TBI [8], with a 2:1 ratio of internal carotid to vertebral artery involvement [34]. The gap of one-to-two orders of magnitude between those figures and our pooled estimates is a direct consequence of the indication filter, since every cohort included here consists of patients in whom a clinician had already decided that vascular imaging was warranted, usually because of a penetrating wound, a skull-base fracture crossing a vascular groove, or a fracture adjacent to a dural sinus. The pooled values therefore describe the diagnostic yield of vascular imaging in selected patients and carry no information about how common these lesions are in unselected TBIs.

The two most frequent mechanisms causing TCVI are blunt and penetrating force. Blunt trauma is characterized by impact with a dull surface or object and commonly occurs in motor vehicle accidents or contact sports, while penetrating trauma refers to injuries caused by objects that pierce the body. This last category includes both high-velocity mechanisms, such as gunshot wounds, and low-velocity mechanisms, such as stab wounds [35].

TCVIs include arterial and venous injuries, and our focus is on intracranial traumatic arterial dissection, arterial pseudoaneurysm, arterial occlusion, venous sinus thrombosis, arterio-venous fistula, and carotid–cavernous fistula.

Pseudoaneurysms were found in 4.8% of imaged patients in the three adult cohorts and in 4.5% when the adult-dominant cohorts were added. Large databases report incidences of traumatic pseudoaneurysms ranging from 0.23% to 0.64%, with higher rates in penetrating injuries [36], and one prospective study reported 12% among 330 patients with head stab wounds who underwent digital subtraction angiography [37]. Our estimate lies between the two, consistent with cohorts selected for angiography after predominantly penetrating trauma.

Heterogeneity is high in both sets (I^2^ = 96.2% in the adult cohorts and 88.7% in the extended set), and the prediction interval for the adult cohorts, ranging from 0.3% to 49.0%, is the honest summary: the next series may report almost any value. In the extended set, univariable meta-regression on imaging modality returned *p* = 0.33, *p* = 0.13 on study design, and *p* = 0.11 on trauma mechanism. Penetrating injury is associated with direct arterial wall damage and pseudoaneurysm formation [38], and the two modalities gave 5.8% with digital subtraction angiography against 2.6% with CT angiography, a difference whose interval is too wide to support or exclude the loss of sensitivity described by Ares et al. [19] and Meyer et al. [25] when CT angiography replaces digital subtraction angiography.

Intracranial arterial occlusion was reported at the patient level by one adult cohort, Lamanna et al. [24], 5.8% (95% CI, 3.2–10.4%), and by two adult-dominant cohorts; the three together gave 12.7% (95% CI, 5.3–27.5%), with estimates between 5.8% and 22.2%. Occlusion is reported at the lesion level by three further cohorts, which is why this analysis rests on 261 patients and why its confidence interval is wide.

Traumatic arterial dissection was reported at the patient level by one adult cohort, Lamanna et al. [24], 7.0% (95% CI, 4.0–11.8%), and the three cohorts of the sensitivity analysis gave 8.3% (95% CI, 5.4–12.4%) with I^2^ = 0%, which on three cohorts and 21 events means only that no between-study variance was detectable. All contributing cohorts consisted of penetrating or mixed trauma, so this review cannot address the association between blunt mechanisms and dissection that is reported in the extracranial literature [39].

Dural venous sinus thrombosis showed the highest pooled diagnostic yield, 11.0% in the four adult cohorts and 17.5% in the ten-cohort set, and the widest spread of individual estimates, ranging from 0.6% to 36.7%, with I^2^ above 90% in both sets. The six cohorts that performed dedicated CT or MR venography reported values ranging from 13.9% to 36.7%, whereas the four that assessed the sinuses on an arterial study reported 0.6%, 4.2%, 12.3%, and 15.9%. In separate exploratory univariable models, trauma mechanism, imaging modality, and study design were each associated with part of the heterogeneity, and the three are confounded here, since five of the six blunt trauma cohorts used dedicated venous imaging and every penetrating-trauma cohort was assessed on an arterial study, so these data cannot say which factor drives the difference, or whether any of them does. The practical consequence is narrow: any screening recommendation for post-traumatic sinus thrombosis has to specify modality and population before it specifies a threshold.

The pooled diagnostic yield of traumatic intracranial arteriovenous fistula was 1.1% (95% CI, 0.0–20.8%) across two adult cohorts and six events, and that of carotid–cavernous fistula was 0.7% (95% CI, 0.2–3.1%) across three adult cohorts and seven events; the extended sets gave 1.4% and 0.8%. Both rest on single-digit event counts, their prediction intervals reach 69.9% and 9.7%, and the lesion-level counts of Meyer et al. [25] and Bodanapally et al. [16] were not converted into patient counts. All contributing cohorts involved penetrating trauma, which matches the direct communication between arterial and venous structures described in published series [40,41].

None of the included cohorts combined vascular imaging with blood-based biomarkers of brain injury. Markers such as glial fibrillary acidic protein, ubiquitin C-terminal hydrolase L1, S100B, neurofilament light chain, and tau can be measured in the acute phase, and the CBI-M framework places them alongside vascular and structural imaging. Prospective cohorts that apply a predefined imaging protocol to a defined trauma population and sample biomarkers at fixed times after injury could test whether biomarker concentrations add to clinical and non-contrast CT variables in predicting an imaging-confirmed vascular lesion. Such work requires standardized collection timing, harmonized assay platforms, and prespecified common outcome measures, none of which can be recovered from the retrospective series that make up the present evidence base.

## 5. Limitations

The pooled estimates apply to adults selected for vascular imaging after traumatic brain injury and cannot be read as population incidences. Every included cohort passed through a clinical indication filter, and in most series, the indication is described only in general terms, so the degree of selection cannot be quantified. Where intracranial and extracranial vascular injuries were not clearly differentiated, only lesions explicitly identified as intracranial were counted, which underestimates the true figure. Eleven of the nineteen cohorts are adult-dominant: they have a mean or median age above 18 years but include a pediatric or adolescent fraction that the source does not allow to be separated, ranging from a lower age bound of 2 years for Delgado Almandoz et al. [13] and 9 years for Meyer et al. [25] to 16 or 17 years for Mansour et al. [23] and Karanja et al. [17], or they state no age criterion at all (Zangbar et al. [15], Dawoud et al. [21], So et al. [20]). These cohorts enter the sensitivity analyses only, and the primary analyses rest on the eight cohorts with an explicit adult criterion or a separately reported adult subgroup (Rivkin et al. [14]). The cost of that restriction is visible in every analysis: two lesions are reported by a single adult cohort and no pooled adult estimate rests on more than four cohorts. The proportion of patients with any lesion is reported cohort by cohort and is not pooled, because the extent of venous assessment and the lesion categories counted differ across the seven cohorts that imaged every patient.

Heterogeneity was high in every pooled primary analysis, with I^2^ between 74.7% and 96.2%, and the prediction intervals span most of the possible range. In the sensitivity analysis, the dissection set showed no detectable heterogeneity, which on three cohorts and 21 events is a statement about statistical power and says nothing about the comparability of the cohorts. Subgroup analyses were possible only in the sensitivity sets, rest on one-to-eight cohorts per group, and are exploratory; for sinus thrombosis, the three moderators are confounded and the available evidence cannot separate them. Meta-regression with more than one covariate was not attempted, since no outcome approached the ten studies per covariate recommended for prevalence data.

The literature search covered PubMed, Web of Science Core Collection, Scopus, and the Cochrane Library. Ninety reports could not be retrieved: 45 sat behind subscriptions the authors could not obtain, 22 were conference abstracts with no full paper, and 23 were scanned or unindexed records without a readable text layer. Ten of the ninety report counts in their abstracts appeared without the full text, the patient-level numerator, the imaging denominator, the intracranial location, or the age range, and the absence of overlap with included cohorts could not be verified, so these reports are described in Table S7 and are not pooled. Eligibility remained limited to English-language full-text articles, which is a residual source of language bias.

Fourteen of the nineteen included studies were retrospective. On the Newcastle–Ottawa Scale, the overall risk of bias was low in ten and moderate in nine; on the Joanna Briggs checklist, twelve were rated at low, six at moderate, and one at high risk of bias. Publication bias was not tested for any outcome, since no primary analysis reached ten studies, and funnel plots are descriptive only.

All counts were re-extracted at the patient level against the source publications, and counts available only at the lesion level were excluded. This removes four cohorts (Bell et al. [12], Meyer et al. [25], Bodanapally et al. [16], and Mansour et al. [23]) from the subtype analyses and leaves one adult cohort each for dissection and occlusion, with three in the sensitivity analyses. Three series were excluded for a denominator not defined by vascular imaging: those of Achram et al. [28], Huang et al. [29], and Kobata et al. [8]. One report identified by the updated search, Bodanapally et al., 2014 [42], was excluded as an overlapping account of the same institutional cohort as Bodanapally et al., 2015 [16].

Clinical outcomes lie outside the scope registered for this review. The protocol deposited on the Open Science Framework (osf.io/thjxp) specifies prevalence, lesion spectrum, and imaging modality as the outcomes of interest, and the eligibility criteria were written to capture cohorts that underwent vascular imaging, with no requirement that outcome data be reported. Mortality, endovascular or surgical treatment, and functional recovery are therefore not synthesized here, and the clinical significance of the lesions described in this review remains undefined by it. A separate synthesis, built on eligibility criteria for prognostic studies, on a risk-of-bias instrument suited to them and on outcomes extracted separately for patients with and without a vascular lesion, is needed to answer that question, and it is the most useful next step in this field. Long-term follow-up capable of detecting neurodegenerative consequences was absent from all included cohorts.

## 6. Conclusions

Traumatic intracranial vascular injuries form a heterogeneous group of arterial and venous lesions. In adults selected for vascular imaging after traumatic brain injury, the proportion with at least one lesion ranges from 1.5% to 50.6% across the three adult cohorts that imaged every patient, according to the indication for imaging and the extent of vascular assessment, and no single figure summarizes it. These values are the diagnostic yield of imaging in selected patients and do not estimate the frequency of vascular lesions among patients with traumatic brain injury in general.

In adult cohorts, dural venous sinus thrombosis was the most frequent single lesion at 11.0%, followed by pseudoaneurysm at 4.8%; arteriovenous and carotid–cavernous fistula remained near 1%, and dissection and occlusion were reported by a single cohort, at 7.0% and 5.8%. Each pooled estimate rests on two-to-four small cohorts, carries very low certainty, and has a prediction interval wide enough that a new cohort may report a value far from it; the confidence intervals of the lesion types overlap, so no rank order among them is supported by these data.

The available evidence comes almost entirely from penetrating trauma for the arterial lesions and from blunt trauma with skull fractures for the venous lesions, with little overlap between the two. Prospective cohorts that apply a predefined imaging protocol to a defined trauma population, report patient-level counts separately for intracranial and cervical lesions, and link them to mortality, treatment, and functional outcome are required before screening thresholds can be proposed.

## Figures and Tables

**Figure 1:**
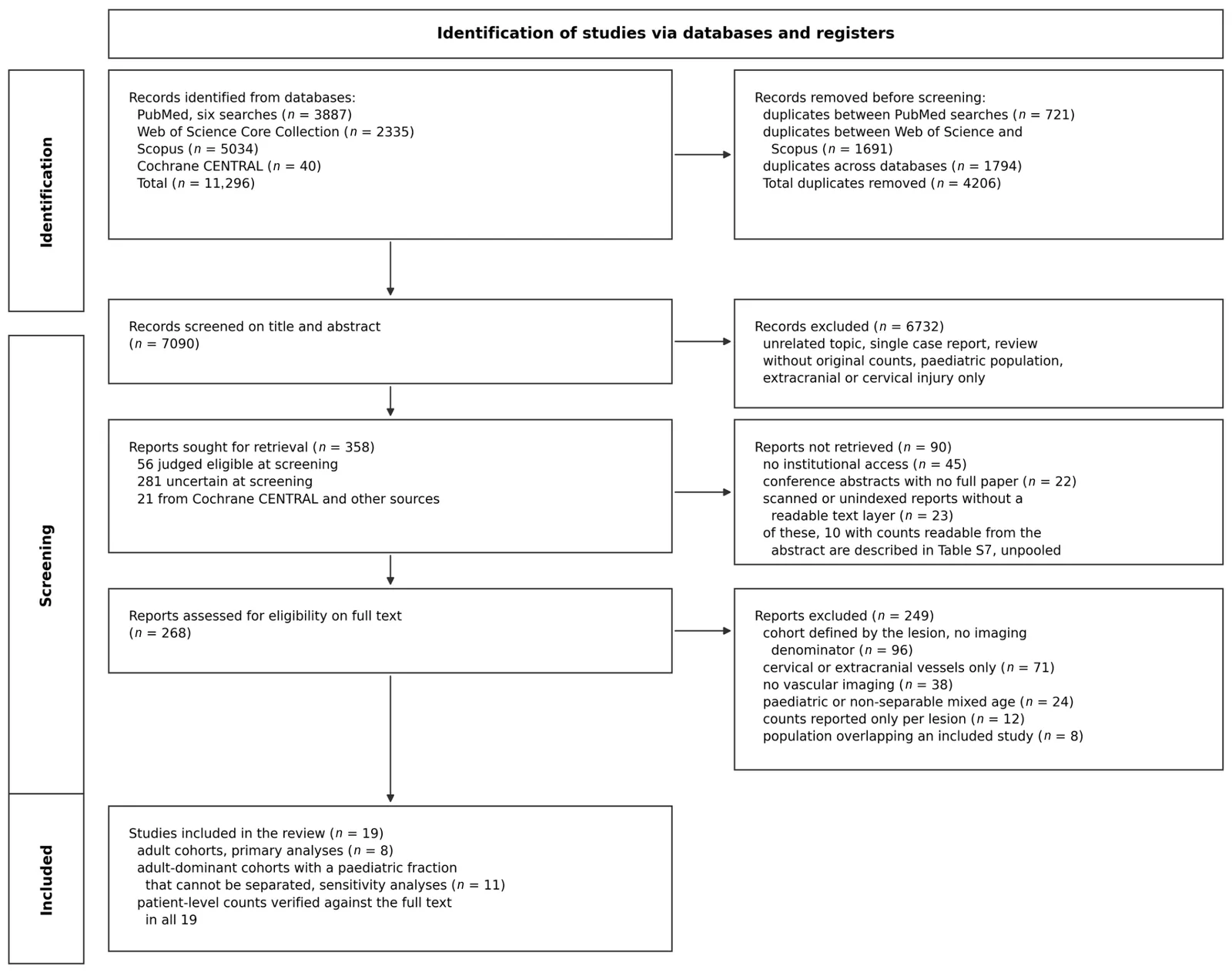
PRISMA flowchart.

**Figure 2:**
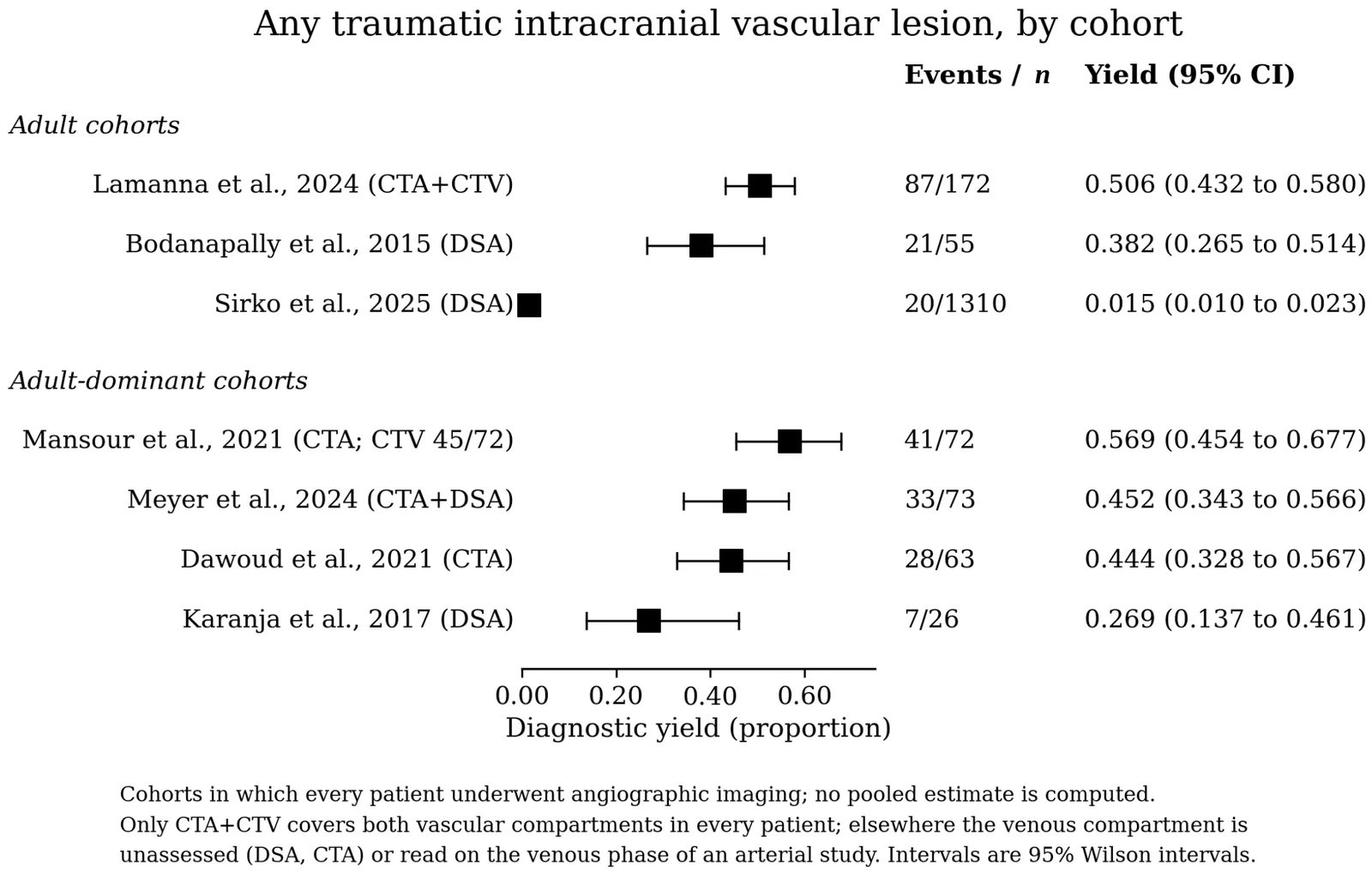
Diagnostic yield of any traumatic intracranial vascular lesion in the seven cohorts in which every patient underwent angiographic imaging (Lamanna et al. [24], Bodanapally et al. [16], Sirko et al. [27], Mansour et al. [23], Meyer et al. [25], Dawoud et al. [21], and Karanja et al. [17]), by cohort and age group, with the imaging protocol in brackets. Squares are cohort estimates and horizontal lines are 95% Wilson intervals. No pooled estimate is computed, because the cohorts differ in whether and how the venous compartment was assessed and in the lesion categories counted (Table S5).

**Table 1:** Characteristics of the nineteen included studies and the counts used in the meta-analyses. Counts are numbers of patients. Cells marked NR indicate a lesion type that the source did not report at the patient level, either because it lay outside the scope of its assessment or because it was reported only as a lesion count; such studies do not enter the analysis of that outcome. Diss., arterial dissection; Pseu., pseudoaneurysm; Occl., arterial occlusion; AVF, arteriovenous fistula; VST, dural venous sinus thrombosis; CCF, carotid–cavernous fistula; Pen., penetrating; Retro., retrospective; Prosp., prospective; NR, not reported; RoB, overall risk of bias on the Joanna Briggs Institute checklist for prevalence studies. Age group (Adult-dom., adult-dominant): Adult, explicit adult inclusion criterion, age range starting at 18 years or separately reported adult subgroup; Adult-dominant, mean or median age above 18 years with a pediatric or adolescent fraction that cannot be separated, or no stated age criterion; adult-dominant cohorts enter the sensitivity analyses only.

Study	Country	Design	Mech.	Imaging	*n*	Any	Diss.	Pseu.	Occl.	AVF	VST	CCF	RoB	Age
Aarabi, 1988 [9]	USA	Retro	Pen	DSA	223	NR	NR	6	NR	NR	NR	NR	Mod	Adult-dom.
Miley, 2008 [10]	USA	Retro	Blunt	DSA	74	NR	NR	4	NR	NR	NR	NR	Mod	Adult-dom.
Ahmed, 2009 [11]	USA	Retro	Blunt	DSA	24	NR	3	4	NR	1	1	NR	Mod	Adult-dom.
Bell, 2010 [12]	USA	Retro, 2 c.	Pen	DSA	187	NR	NR	24	NR	NR	NR	NR	Low	Adult
Delgado A., 2010 [13]	USA	Retro	Blunt	CTV	195	NR	NR	NR	NR	NR	57	NR	Low	Adult-dom.
Rivkin, 2014 [14]	USA	Retro	Blunt	CTV	49	NR	NR	NR	NR	NR	18	NR	Mod	Adult
Zangbar, 2015 [15]	USA	Retro	Both	CTA	459	NR	NR	3	NR	NR	NR	NR	Low	Adult-dom.
Bodanapally, 2015 [16]	USA	Retro	Pen	DSA	55	21	NR	NR	NR	NR	NR	NR	Low	Adult
Karanja, 2017 [17]	S. Africa	Prosp/retro	Pen	CTA + DSA	26	7	NR	3	4	NR	NR	NR	Mod	Adult-dom.
Slasky, 2017 [18]	USA	Retro	Blunt	CTV	210	NR	NR	NR	NR	NR	48	NR	Low	Adult-dom.
Ares, 2019 [19]	USA	Retro	Pen	CTA + DSA	56	NR	NR	NR	NR	3	NR	NR	Low	Adult
So, 2019 [20]	Australia	Retro	Blunt	CTV	102	NR	NR	NR	NR	NR	37	NR	Low	Adult-dom.
Dawoud, 2021 [21]	USA	Retro	Pen	CTA	63	28	6	2	14	0	10	NR	Low	Adult-dom.
Harrington, 2021 [22]	S. Africa	Prosp	Pen	CTA + DSA	156	NR	NR	NR	NR	NR	1	2	Low	Adult
Mansour, 2021 [23]	USA	Retro	Pen	CTA + CTV	72	41	NR	NR	NR	NR	NR	1	Mod	Adult-dom.
Lamanna, 2024 [24]	USA	Retro	Pen	CTA + CTV	172	87	12	12	10	NR	27	3	Low	Adult
Meyer, 2024 [25]	USA	Prosp, 2 c.	Pen	CTA + DSA	73	33	NR	NR	NR	NR	9	NR	Low	Adult-dom.
McGuckin, 2024 [26]	Australia	Prosp	Blunt	CTV/MRV	36	NR	NR	NR	NR	NR	5	NR	Mod	Adult
Sirko, 2025 [27]	Ukraine	Prosp	Pen	DSA	1310	20	NR	15	NR	3	NR	2	Low	Adult
Total					3542									

## Data Availability

The original contributions presented in this study are included in the article/Supplementary Materials. Further inquiries can be directed to the corresponding authors.

## Supplementary Materials

The following supporting information can be downloaded at https://www.mdpi.com/article/10.3390/medicina62101830/s1, Table S1: Newcastle–Ottawa Scale ratings; Table S2: Joanna Briggs Institute checklist ratings; Table S3: GRADE certainty of evidence; Table S4: exploratory univariable meta-regressions; Table S5: imaging protocols and lesion categories of the cohorts reporting any lesion; Table S6: primary and sensitivity estimates side by side; Table S7: reports with counts readable only from the abstract; Table S8: PRISMA 2020 checklist; Figures S1 and S2: funnel plots; Reference [43] is mentioned in Table S8.

## Abbreviations

The following abbreviations are used in this manuscript:

TBI	Traumatic brain injury
TCVI	Traumatic cerebrovascular injury
MRI	Magnetic resonance imaging
CT	Computed tomography
CTA	Computed tomographic angiography
MRA	Magnetic resonance angiography
CTV	Computed tomographic venography
MRV	Magnetic resonance venography
DSA	Digital subtraction angiography
AVF	Arteriovenous fistula
CCF	Carotid–cavernous fistula
