# Supplementary material for: Traumatic Intracranial Vascular Lesions in Adults Undergoing Vascular Imaging After Traumatic Brain Injury: A Systematic Review and Meta-Analysis
Source: Medicina (Kaunas). 2026 Sep 23;62(10):1830. doi: 10.3390/medicina62101830 (PMC13706739; doi:10.3390/medicina62101830)
Supplement: Supplementary file 1 [file medicina-62-01830-s001.zip › Supplementary Materials.pdf]

Supplementary Materials

Supplementary S1. Search strategies

All four databases were searched on 31 August 2026, from database inception, with no date, language or age restriction. PubMed was searched with six queries, each combining the same trauma block with one lesion block; the number of records returned by each is given below. Records were deduplicated on PubMed identifier within PubMed, then on DOI, PubMed identifier and normalised title with year across databases.

PubMed. Trauma block, identical in all six queries:

```
("Craniocerebral Trauma"[MeSH] OR "traumatic brain injury"[tiab] OR "head injury"[tiab] OR "head trauma"[tiab])
```

The MeSH descriptor Craniocerebral Trauma explodes to include Brain Injuries Traumatic, Head Injuries Penetrating and Skull Fractures. Exclusion filter, applied in queries 1 to 5:

```
NOT ("Editorial"[pt] OR "Letter"[pt] OR "Comment"[pt] OR "News"[pt])
```

Query 1. Aneurysm and pseudoaneurysm, 947 records.

```
TRAUMA BLOCK AND ("Aneurysm, False"[MeSH] OR "pseudoaneurysm"[tiab] OR "pseudoaneurysms"[tiab] OR "traumatic aneurysm"[tiab] OR "traumatic aneurysms"[tiab] OR "intracranial aneurysm"[tiab] OR "intracranial aneurysms"[tiab]) NOT FILTER
```

Query 2. Arterial dissection and occlusion, 275 records.

```
TRAUMA BLOCK AND ("Vertebral Artery Dissection"[MeSH] OR "arterial dissection"[tiab] OR "artery dissection"[tiab] OR "arterial occlusion"[tiab] OR "vessel occlusion"[tiab]) NOT FILTER
```

Query 3. Dural venous sinus thrombosis, 369 records.

```
TRAUMA BLOCK AND ("Sinus Thrombosis, Intracranial"[MeSH] OR "venous sinus thrombosis"[tiab] OR "dural sinus thrombosis"[tiab] OR "sinus thrombosis"[tiab] OR "venous sinus injury"[tiab]) NOT FILTER
```

Query 4. Arteriovenous and carotid-cavernous fistula, 627 records.

```
TRAUMA BLOCK AND ("Carotid-Cavernous Sinus Fistula"[MeSH] OR "arteriovenous fistula"[tiab] OR "arteriovenous fistulas"[tiab] OR "carotid-cavernous fistula"[tiab] OR "carotid cavernous fistula"[tiab]) NOT FILTER
```

Query 5. Cerebrovascular injury, general terms, 1575 records.

```
TRAUMA BLOCK AND ("Cerebrovascular Trauma"[MeSH] OR "Carotid Artery Injuries"[MeSH] OR "cerebrovascular injury"[tiab] OR "cerebrovascular injuries"[tiab] OR "vascular injury"[tiab] OR "vascular injuries"[tiab]) NOT FILTER
```

Query 6. Diagnostic yield of vascular imaging, 94 records, no publication-type filter.

```
TRAUMA BLOCK AND ("digital subtraction angiography"[tiab] OR "CT angiography"[tiab] OR "computed tomographic angiography"[tiab] OR "CT venography"[tiab] OR "MR venography"[tiab]) AND ("screening"[tiab] OR "yield"[tiab] OR "prevalence"[tiab] OR "incidence"[tiab])
```

The six queries returned 3887 records, of which 3166 were unique.

Web of Science Core Collection, 2335 records.

```
TS=("traumatic brain injury" OR "head injury" OR "head injuries" OR "head trauma" OR "craniocerebral trauma" OR "penetrating head injur*" OR "penetrating brain injur*" OR "gunshot wound*" OR "stab wound*" OR "missile injur*" OR "skull fracture*")
AND TS=("pseudoaneurysm*" OR "false aneurysm*" OR "traumatic aneurysm*" OR "traumatic intracranial aneurysm*" OR "intracranial aneurysm*" OR "arterial dissection*" OR "artery dissection*" OR "arterial occlusion*" OR "vessel occlusion*" OR "vessel cut-off" OR "venous sinus thrombosis" OR "dural sinus thrombosis" OR "sinus thrombosis" OR "venous sinus injur*" OR "arteriovenous fistula*" OR "carotid-cavernous fistula*" OR "carotid cavernous fistula*" OR "cerebrovascular injur*" OR "cerebrovascular trauma" OR "vascular injur*")
NOT DT=("Editorial Material" OR "Letter" OR "News Item" OR "Correction")
```

Scopus, 5034 records.

```
TITLE-ABS-KEY("traumatic brain injury" OR "head injury" OR "head injuries" OR "head trauma" OR "craniocerebral trauma" OR "penetrating head injury" OR "penetrating brain injury" OR "gunshot wound*" OR "stab wound*" OR "missile injur*" OR "skull fracture*")
AND TITLE-ABS-KEY("pseudoaneurysm*" OR "false aneurysm*" OR "traumatic aneurysm*" OR "intracranial aneurysm*" OR "arterial dissection*" OR "artery dissection*" OR "arterial occlusion*" OR "vessel occlusion*" OR "vessel cut-off" OR "venous sinus thrombosis" OR "dural sinus thrombosis" OR "sinus thrombosis" OR "venous sinus injur*" OR "arteriovenous fistula*" OR "carotid-cavernous fistula*" OR "carotid cavernous fistula*" OR "cerebrovascular injur*" OR "cerebrovascular trauma" OR "vascular injur*")
AND NOT DOCTYPE(ed) AND NOT DOCTYPE(le) AND NOT DOCTYPE(no) AND NOT DOCTYPE(er)
```

Scopus indexes the South African Journal of Radiology and retrieved Karanja et al. 2017, which has no PubMed record.

Cochrane Library, CENTRAL, 40 records.

```
#1 MeSH descriptor: [Craniocerebral Trauma] explode all trees
#2 ("traumatic brain injury" OR "head injury" OR "head injuries" OR "head trauma" OR "penetrating head injury" OR "gunshot wound" OR "stab wound" OR "skull fracture"):ti,ab,kw
#3 #1 OR #2
#4 MeSH descriptor: [Aneurysm, False] explode all trees
#5 MeSH descriptor: [Sinus Thrombosis, Intracranial] explode all trees
#6 MeSH descriptor: [Carotid-Cavernous Sinus Fistula] explode all trees
#7 MeSH descriptor: [Cerebrovascular Trauma] explode all trees
#8 (pseudoaneurysm OR "traumatic aneurysm" OR "intracranial aneurysm" OR "arterial dissection" OR "arterial occlusion" OR "venous sinus thrombosis" OR "dural sinus thrombosis" OR "arteriovenous fistula" OR "carotid-cavernous fistula" OR "cerebrovascular injury" OR "vascular injury"):ti,ab,kw
#9 #4 OR #5 OR #6 OR #7 OR #8
#10 #3 AND #9
```

The search returned 40 trial records and no Cochrane review or protocol.

Table S1. Risk of bias, Newcastle-Ottawa Scale

Study	Year	Country	Design	Overall risk of bias
Aarabi	1988	USA	Retrospective	Moderate
Miley et al.	2008	USA	Retrospective	Moderate
Ahmed et al.	2009	USA	Retrospective	Moderate
Bell et al.	2010	USA	Retrospective, two centres	Low
Delgado Almandoz et al.	2010	USA	Retrospective	Low
Rivkin et al.	2014	USA	Retrospective	Moderate
Zangbar et al.	2015	USA	Retrospective	Low
Bodanapally et al.	2015	USA	Retrospective	Low
Karanja et al.	2017	South Africa	Prospective enrolment, retrospective review	Moderate
Slasky et al.	2017	USA	Retrospective	Low
Ares et al.	2019	USA	Retrospective	Low
So et al.	2019	Australia	Retrospective	Moderate
Dawoud et al.	2021	USA	Retrospective	Low
Harrington et al.	2021	South Africa	Prospective	Low
Mansour et al.	2021	USA	Retrospective	Low
Lamanna et al.	2024	USA	Retrospective	Low
Meyer et al.	2024	USA	Prospective, two centres	Low
McGuckin et al.	2024	Australia	Prospective	Moderate
Sirko et al.	2025	Ukraine	Prospective	Low

Assessed independently by two reviewers on selection, comparability and outcome ascertainment, with disagreements resolved by consensus. Ten studies were rated at low and nine at moderate overall risk of bias; none was rated at high risk.

Table S2. Risk of bias, Joanna Briggs Institute checklist for prevalence studies

Study	Q1	Q2	Q3	Q4	Q5	Q6	Q7	Q8	Q9	Yes	No	Unclear	Overall
Aarabi	Y	U	Y	Y	Y	Y	Y	U	NA	6	0	2	Moderate
Miley et al.	Y	U	U	Y	Y	Y	Y	U	NA	5	0	3	Moderate
Ahmed et al.	Y	U	N	Y	U	Y	Y	Y	NA	5	1	2	Moderate
Bell et al.	Y	Y	Y	Y	Y	Y	Y	U	NA	7	0	1	Low
Delgado Almandoz et al.	Y	U	Y	Y	Y	Y	Y	Y	NA	7	0	1	Low
Rivkin et al.	Y	U	N	Y	Y	Y	Y	Y	NA	6	1	1	Moderate
Zangbar et al.	Y	Y	Y	Y	Y	Y	Y	Y	NA	8	0	0	Low
Bodanapally et al.	Y	Y	U	Y	Y	Y	Y	Y	NA	7	0	1	Low
Karanja et al.	Y	U	N	Y	Y	Y	Y	Y	U	6	1	2	Moderate
Slasky et al.	Y	Y	Y	Y	Y	Y	Y	Y	NA	8	0	0	Low
Ares et al.	Y	Y	U	Y	Y	Y	Y	Y	NA	7	0	1	Low
So et al.	Y	U	Y	Y	Y	Y	Y	Y	NA	7	0	1	Low
Dawoud et al.	Y	Y	U	Y	Y	Y	Y	Y	NA	7	0	1	Low
Harrington et al.	Y	Y	Y	Y	Y	U	Y	Y	Y	8	0	1	Low
Mansour et al.	Y	Y	U	Y	Y	Y	N	Y	NA	6	1	1	Moderate
Lamanna et al.	Y	Y	Y	Y	Y	Y	Y	Y	NA	8	0	0	Low
Meyer et al.	Y	Y	U	Y	Y	Y	Y	Y	Y	8	0	1	Low
McGuckin et al.	Y	Y	N	Y	Y	Y	U	Y	Y	7	1	1	Moderate
Sirko et al.	Y	Y	Y	Y	Y	Y	Y	Y	Y	9	0	0	Low

Q1, sampling frame appropriate; Q2, sampling method appropriate; Q3, sample size adequate; Q4, subjects and setting described; Q5, coverage of the analysis adequate; Q6, valid methods used to identify the condition; Q7, reliable measurement of the condition; Q8, appropriate statistical analysis; Q9, response rate adequate. Y, yes; N, no; U, unclear; NA, not applicable. Overall risk of bias: low (at least seven yes and no no), moderate (five or six yes, or at least seven yes with one no), high (four or fewer yes, or two or more no). Twelve studies were rated at low and six at moderate risk of bias; the Joanna Briggs judgement was used as the risk-of-bias input to the GRADE assessment.

Table S3. Certainty of the evidence, GRADE adapted for prevalence

Outcome	k	Events/patients	Yield % (95% CI)	Prediction interval %	I2 %	Risk of bias	Inconsistency	Indirectness	Imprecision	Publication bias	Certainty
Dural venous sinus thrombosis	4	51/413	11.0 (2.3 to 39.8)	0.3 to 81.7	95.1	Serious	Very serious	Serious	Very serious	Not assessable (k < 10)	Very low
Arterial occlusion	1	10/172	5.8 (3.2 to 10.4), single cohort	Not applicable	Not applicable	Serious	Not assessable (one cohort)	Serious	Very serious	Not assessable	Very low
Arterial dissection	1	12/172	7.0 (4.0 to 11.8), single cohort	Not applicable	Not applicable	Serious	Not assessable (one cohort)	Serious	Very serious	Not assessable	Very low
Pseudoaneurysm	3	51/1669	4.8 (1.1 to 18.4)	0.3 to 49.0	96.2	Serious	Very serious	Serious	Very serious	Not assessable (k < 10)	Very low
Arteriovenous fistula	2	6/1366	1.1 (0.0 to 20.8)	0.0 to 69.9	93.3	Serious	Very serious	Serious	Very serious	Not assessable (k < 10)	Very low
Carotid-cavernous fistula	3	7/1638	0.7 (0.2 to 3.1)	0.0 to 9.7	74.7	Serious	Serious	Serious	Very serious	Not assessable (k < 10)	Very low

GRADE was applied to the six lesion-specific outcomes of the primary analysis, which uses the eight adult cohorts; the proportion of patients with any lesion was not pooled and is not rated. Certainty starts at moderate for bodies of observational prevalence evidence and is downgraded by domain. Risk of bias is serious throughout, since most cohorts are retrospective and every cohort was assembled on a clinical indication for imaging. Indirectness is serious for the same reason, because the question concerns adults with traumatic brain injury while the evidence describes the fraction of them selected for imaging. Inconsistency follows I2, rated not serious below 50%, serious between 50 and 75% and very serious above 75%. Imprecision is very serious where the confidence interval spans more than 20 percentage points or fewer than five cohorts contribute, and serious where fewer than 30 events are available. Two outcomes, arterial dissection and arterial occlusion, are reported by a single adult cohort and are rated on that cohort alone.

Table S4. Exploratory univariable meta-regressions, sensitivity sets

**Analysis (all cohorts)**	**Subgroup**	**k**	**Yield % (95% CI)**	**p, univariable meta-regression**	**Heterogeneity associated with the moderator, %**
Dural venous sinus thrombosis, by imaging modality	Arterial study only (CTA, CTA+DSA)	4	6.2 (1.6 to 21.1)	0.006	54
Dural venous sinus thrombosis, by imaging modality	Dedicated venous imaging (CTV, MRV)	6	25.4 (18.6 to 33.7)	0.006	54
Dural venous sinus thrombosis, by study design	Prospective	3	6.0 (1.0 to 28.7)	0.019	50
Dural venous sinus thrombosis, by study design	Retrospective	7	24.0 (17.3 to 32.2)	0.019	50
Dural venous sinus thrombosis, by trauma mechanism	Blunt	6	26.7 (19.9 to 35.0)	0.008	73
Dural venous sinus thrombosis, by trauma mechanism	Penetrating	4	9.0 (2.9 to 24.8)	0.008	73
Pseudoaneurysm, by imaging modality	CT angiography	3	2.6 (0.6 to 10.3)	0.329	0
Pseudoaneurysm, by imaging modality	Digital subtraction angiography	6	5.8 (2.4 to 13.4)	0.329	0
Pseudoaneurysm, by study design	Prospective	1	1.1 (0.7 to 1.9)	0.130	19
Pseudoaneurysm, by study design	Retrospective	8	5.5 (2.7 to 11.0)	0.130	19
Pseudoaneurysm, by trauma mechanism	Blunt	2	9.5 (3.0 to 26.5)	0.113	24
Pseudoaneurysm, by trauma mechanism	Mixed	1	0.7 (0.2 to 2.0)	0.113	24
Pseudoaneurysm, by trauma mechanism	Penetrating	6	4.7 (2.1 to 10.3)	0.113	24

Moderators were examined only in the sensitivity sets, which combine adult and adult-dominant cohorts, because the primary sets have two to four cohorts. Each moderator was fitted alone, by univariable meta-regression with a single residual between-study variance, with every cohort of the analysis entered. Imaging modality was recoded on two levels defined by what the protocol can detect: for dural venous sinus thrombosis, dedicated venous imaging (CT or MR venography, alone or with CT angiography) against an arterial study (CT angiography, with or without digital subtraction angiography); for the arterial outcomes, a protocol including digital subtraction angiography against CT angiography alone. The last column is the proportion of between-study variance associated with the moderator in its own univariable model; the values of different moderators come from separate models and cannot be added, compared or read as independent contributions. For dural venous sinus thrombosis the three moderators are confounded, since five of the six blunt-trauma cohorts used dedicated venous imaging and every penetrating-trauma cohort was assessed on an arterial study. Every comparison rests on one to eight cohorts per group and is exploratory.

Table S5. Imaging protocols and lesion categories of the cohorts reporting the number of patients with any lesion

**Study**	**Age group**	**Imaging protocol**	**Venous compartment**	**Lesion categories in the numerator**	**Events/patients**	**Yield % (95% Wilson CI)**

Lamanna 2024	Adult	CT angiography and CT venography in all 172	Dedicated CT venography in every patient	Intracranial arterial and venous lesions, including sinus impingement (7) and transection (6)	87/172	50.6 (43.2 to 58)
Bodanapally 2015	Adult	Digital subtraction angiography in all 55	Not assessed	Intracranial arterial lesions	21/55	38.2 (26.5 to 51.4)
Sirko 2025	Adult	Digital subtraction angiography in all 1310 head and neck wounds	Not assessed	Intracranial arterial lesions	20/1310	1.5 (1 to 2.3)
Mansour 2021	Adult-dominant	CT angiography in all 72; CT venography in 45 on clinical suspicion	Partial, on suspicion (45 of 72)	Arterial lesions; venous lesions in the 45 with venography	41/72	56.9 (45.4 to 67.7)
Meyer 2024	Adult-dominant	CT angiography and digital subtraction angiography in all 73	Venous phase of the arterial study	Pseudoaneurysm, major arterial occlusion, dural sinus occlusion, dural AVF, CCF	33/73	45.2 (34.3 to 56.6)
Dawoud 2021	Adult-dominant	CT angiography in all 63	Venous phase of the arterial study	Arterial occlusion, dissection, pseudoaneurysm, venous thrombosis, vasospasm (9) and transection (3)	28/63	44.4 (32.8 to 56.7)
Karanja 2017	Adult-dominant	Unilateral carotid digital subtraction angiography in all 26	Not assessed	Pseudoaneurysm and vessel cut-off	7/26	26.9 (13.7 to 46.1)

These seven cohorts applied angiographic imaging to every patient. Only Lamanna et al. assessed the venous compartment by dedicated venography in every patient; Meyer et al. and Dawoud et al. read the dural sinuses on the venous phase of an arterial study; Mansour et al. performed venography in 45 of 72 patients on clinical suspicion; the remaining three did not assess the venous compartment. Two sources count lesion categories outside the six of this review in their numerator. For these reasons the yields are reported cohort by cohort and are not pooled.

Table S6. Primary analysis (adult cohorts) and sensitivity analysis (adult and adult-dominant cohorts) side by side

Outcome	Adult cohorts, k	Adult cohorts, events/patients	Adult cohorts, yield % (95% CI)	Adult cohorts, I2 %	All cohorts, k	All cohorts, events/patients	All cohorts, yield % (95% CI)	All cohorts, prediction interval %	All cohorts, I2 %
Dural venous sinus thrombosis	4	51/413	11.0 (2.3 to 39.8)	95.1	10	213/1080	17.5 (10.8 to 27.2)	3.8 to 53.4	90.4
Arterial occlusion	1	10/172	5.8 (3.2 to 10.4), single cohort	Not applicable	3	28/261	12.7 (5.3 to 27.5)	2.5 to 45.7	80.3
Arterial dissection	1	12/172	7.0 (4.0 to 11.8), single cohort	Not applicable	3	21/259	8.3 (5.4 to 12.4)	5.4 to 12.4	0.0
Pseudoaneurysm	3	51/1669	4.8 (1.1 to 18.4)	96.2	9	73/2538	4.5 (2.2 to 9.2)	0.5 to 30.7	88.7
Arteriovenous fistula	2	6/1366	1.1 (0.0 to 20.8)	93.3	4	7/1453	1.4 (0.3 to 7.2)	0.1 to 28.3	77.1
Carotid-cavernous fistula	3	7/1638	0.7 (0.2 to 3.1)	74.7	4	8/1710	0.8 (0.3 to 2.6)	0.1 to 6.8	62.4

Adult cohorts have an explicit inclusion criterion of 18 years or over, an age range starting at 18 years or a separately reported adult subgroup (Bell, Bodanapally, Lamanna, Ares, McGuckin, Sirko, Harrington; Rivkin with its adult subgroup). Adult-dominant cohorts have a mean or median age above 18 years with a paediatric or adolescent fraction that cannot be separated, or no stated age criterion (Aarabi, Zangbar, Miley, Ahmed, Delgado Almandoz, Slasky, Karanja, So, Dawoud, Mansour, Meyer). Outcomes reported by a single adult cohort are given as that cohort's proportion with its Wilson interval.

Table S7. Reports with counts readable only from the abstract, described and not pooled

Study	Lesion reported	Counts read from the abstract	Modality stated	Mechanism	Not verifiable without the full text
Wang 2013	Dural venous sinus thrombosis	221/403	CTV	Blunt	Patient-level numerator; imaging denominator; intracranial location; age range; overlap with included cohorts
Kim 2022	Dural venous sinus thrombosis	137/424	CTV	Blunt	Patient-level numerator; imaging denominator; intracranial location; age range; overlap with included cohorts

Ma 2024	Dural venous sinus thrombosis	28/61	CTV	Blunt	Patient-level numerator; imaging denominator; intracranial location; age range; overlap with included cohorts
Gabay 2025	Dural venous sinus thrombosis	31/95	CTV	Blunt	Patient-level numerator; imaging denominator; intracranial location; age range; overlap with included cohorts
Stanisce 2024	Dural venous sinus thrombosis	37/220	CTV	Blunt	Patient-level numerator; imaging denominator; intracranial location; age range; overlap with included cohorts
Hendrickson 2014	Dural venous sinus thrombosis	14/29	CTV	Blunt	Patient-level numerator; imaging denominator; intracranial location; age range; overlap with included cohorts
Fujii 2009	Dural venous sinus thrombosis	22/97	CTV	Blunt	Patient-level numerator; imaging denominator; intracranial location; age range; overlap with included cohorts
Li 2015	Dural venous sinus thrombosis	40/240	CTV/MRV	Blunt	Patient-level numerator; imaging denominator; intracranial location; age range; overlap with included cohorts
Chen 2024	Arterial occlusion	26/358	CTA	Blunt	Patient-level numerator; imaging denominator; intracranial location; age range; overlap with included cohorts
Shi 2003	Pseudoaneurysm	6/32	DSA	Blunt	Patient-level numerator; imaging denominator; intracranial location; age range; overlap with included cohorts

These ten reports were identified by the updated search and their abstracts state a numerator and a denominator, but no full text could be obtained. Full text availability is an eligibility criterion, and without it the patient-level nature of the numerator, the imaging denominator, the intracranial location of the lesions, the age range and the absence of overlap with included cohorts cannot be verified. They are therefore listed here and enter no quantitative analysis.

Table S8. PRISMA 2020 checklist

Section and Topic	Item #	Checklist item	Location where item is reported
**TITLE**			
Title	1	Identify the report as a systematic review.	Page 1
**ABSTRACT**			
Abstract	2	See the PRISMA 2020 for Abstracts checklist.	Page 1-2
**INTRODUCTION**			
Rationale	3	Describe the rationale for the review in the context of existing knowledge.	Page 2-3, Introduction, paragraphs 1-4
Objectives	4	Provide an explicit statement of the objective(s) or question(s) the review addresses.	Page 3, Introduction, final paragraph
**METHODS**			
Eligibility criteria	5	Specify the inclusion and exclusion criteria for the review and how studies were grouped for the syntheses.	Page 4, section 2.3; Page 5,

Section and Topic	Item #	Checklist item	Location where item is reported
			section 2.5
Information sources	6	Specify all databases, registers, websites, organisations, reference lists and other sources searched or consulted to identify studies. Specify the date when each source was last searched or consulted.	Page 3, section 2.2
Search strategy	7	Present the full search strategies for all databases, registers and websites, including any filters and limits used.	Page 3, section 2.2
Selection process	8	Specify the methods used to decide whether a study met the inclusion criteria of the review, including how many reviewers screened each record and each report retrieved, whether they worked independently, and if applicable, details of automation tools used in the process.	Page 4,5
Data collection process	9	Specify the methods used to collect data from reports, including how many reviewers collected data from each report, whether they worked independently, any processes for obtaining or confirming data from study investigators, and if applicable, details of automation tools used in the process.	Page 4, section 2.4
Data items	10a	List and define all outcomes for which data were sought. Specify whether all results that were compatible with each outcome domain in each study were sought (e.g. for all measures, time points, analyses), and if not, the methods used to decide which results to collect.	Page 4, section 2.4, Page 4-5, section 2.5
	10b	List and define all other variables for which data were sought (e.g. participant and intervention characteristics, funding sources). Describe any assumptions made about any missing or unclear information.	Page 4, section 2.4
Study risk of bias assessment	11	Specify the methods used to assess risk of bias in the included studies, including details of the tool(s) used, how many reviewers assessed each study and whether they worked independently, and if applicable, details of automation tools used in the process.	Page 5, section 2.6
Effect measures	12	Specify for each outcome the effect measure(s) (e.g. risk ratio, mean difference) used in the synthesis or presentation of results.	Page 4-5, section 2.5
Synthesis methods	13a	Describe the processes used to decide which studies were eligible for each synthesis (e.g. tabulating the study intervention characteristics and comparing against the planned groups for each synthesis (item #5)).	Page 5, section 2.5
	13b	Describe any methods required to prepare the data for presentation or synthesis, such as handling of missing summary statistics, or data conversions.	Page 4, section 2.4; Page 9, section 3.2
	13c	Describe any methods used to tabulate or visually display results of individual studies and syntheses.	Page 7-8 (Table 1); Page 10-15 (Figures 2-8, forest plots)
	13d	Describe any methods used to synthesize results and provide a rationale for the choice(s). If meta-analysis was performed, describe the model(s), method(s) to identify the presence and extent of statistical heterogeneity, and software package(s) used.	Page 5, section 2.5
	13e	Describe any methods used to explore possible causes of heterogeneity among study results (e.g. subgroup analysis, meta-regression).	Page 5, section 2.5; Page 15-16, section 3.4
	13f	Describe any sensitivity analyses conducted to assess robustness of the synthesized results.	Page 5
Reporting bias assessment	14	Describe any methods used to assess risk of bias due to missing results in a synthesis (arising from reporting biases).	Page 5, section 2.5

Section and Topic	Item #	Checklist item	Location where item is reported
Certainty assessment	15	Describe any methods used to assess certainty (or confidence) in the body of evidence for an outcome.	Page 5, section 2.6
**RESULTS**			
Study selection	16a	Describe the results of the search and selection process, from the number of records identified in the search to the number of studies included in the review, ideally using a flow diagram.	Pages 5-6, section 3.1; Page 3, Figure 1
	16b	Cite studies that might appear to meet the inclusion criteria, but which were excluded, and explain why they were excluded.	Page 6,9, Table S7
Study characteristics	17	Cite each included study and present its characteristics.	Pages 7-8, Table 1; Page 9, section 3.2
Risk of bias in studies	18	Present assessments of risk of bias for each included study.	Pages 7-8, Table 1; Page 19; Tables S1-S2
Results of individual studies	19	For all outcomes, present, for each study: (a) summary statistics for each group (where appropriate) and (b) an effect estimate and its precision (e.g. confidence/credible interval), ideally using structured tables or plots.	Pages 7-8, Table 1; Pages 10,11,12,13,14,15, Figures 2-8
Results of syntheses	20a	For each synthesis, briefly summarise the characteristics and risk of bias among contributing studies.	Pages 9-15
	20b	Present results of all statistical syntheses conducted. If meta-analysis was done, present for each the summary estimate and its precision (e.g. confidence/credible interval) and measures of statistical heterogeneity. If comparing groups, describe the direction of the effect.	Pages 10,11,12,13,14,15, figures 3-8
	20c	Present results of all investigations of possible causes of heterogeneity among study results.	Pages 15-16, section 3.4
	20d	Present results of all sensitivity analyses conducted to assess the robustness of the synthesized results.	Pages 10,11,12,13,14,15
Reporting biases	21	Present assessments of risk of bias due to missing results (arising from reporting biases) for each synthesis assessed.	Page 15
Certainty of evidence	22	Present assessments of certainty (or confidence) in the body of evidence for each outcome assessed.	Page 5, section 2.6; Pages 19-20
**DISCUSSION**			
Discussion	23a	Provide a general interpretation of the results in the context of other evidence.	Pages 16-18, section 4.
	23b	Discuss any limitations of the evidence included in the review.	Pages 18-19, section 5
	23c	Discuss any limitations of the review processes used.	Pages 18-19, section 5

Section and Topic	Item #	Checklist item	Location where item is reported
	23d	Discuss implications of the results for practice, policy, and future research.	Pages 19-20, section 6
**OTHER INFORMATION**			
Registration and protocol	24a	Provide registration information for the review, including register name and registration number, or state that the review was not registered.	Page 3, section 2.1
	24b	Indicate where the review protocol can be accessed, or state that a protocol was not prepared.	Page 3, section 2.1; Page 19, section 5
	24c	Describe and explain any amendments to information provided at registration or in the protocol.	Not reported
Support	25	Describe sources of financial or non-financial support for the review, and the role of the funders or sponsors in the review.	Page 20
Competing interests	26	Declare any competing interests of review authors.	Page 20
Availability of data, code and other materials	27	Report which of the following are publicly available and where they can be found: template data collection forms; data extracted from included studies; data used for all analyses; analytic code; any other materials used in the review.	Page 20

From: Page MJ, McKenzie JE, Bossuyt PM, Boutron I, Hoffmann TC, Mulrow CD, et al. The PRISMA 2020 statement: an updated guideline for reporting systematic reviews. BMJ 2021;372:n71. doi: 10.1136/bmj.n71. This work is licensed under CC BY 4.0. To view a copy of this license, visit https://creativecommons.org/licenses/by/4.0/

Figures S1 and S2. Funnel plots

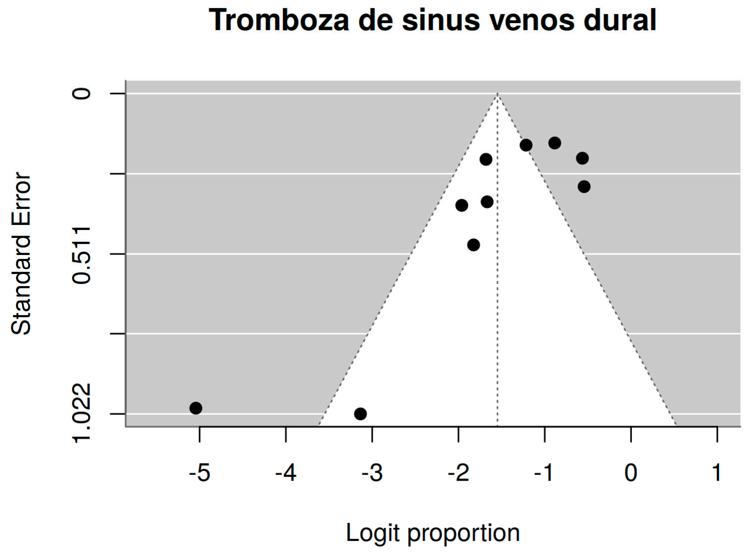

Figure S1. Funnel plot for the ten cohorts of the sensitivity set reporting dural venous sinus thrombosis, on the logit scale. The solid vertical line is the pooled random-effects estimate and the dashed lines are pseudo 95% confidence contours. No primary analysis reached the ten studies conventionally required for a formal test, so the plot is descriptive.

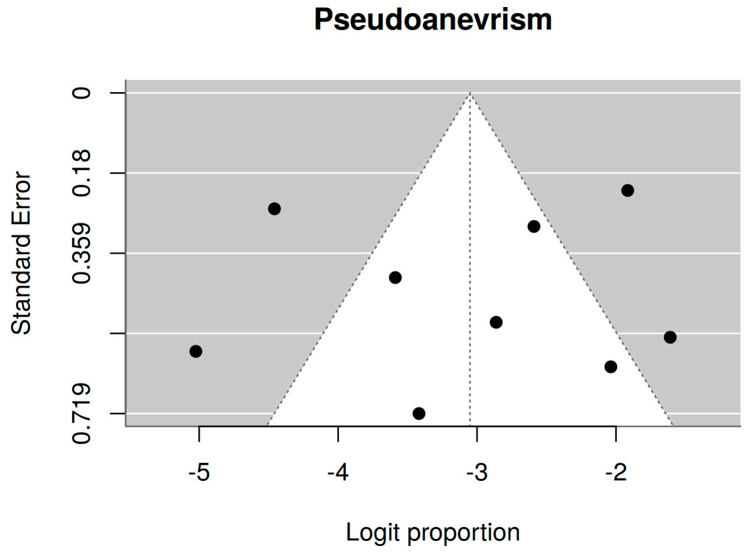

Figure S2. Funnel plot for the nine cohorts of the sensitivity set reporting traumatic intracranial pseudoaneurysm, on the logit scale, descriptive only.
